# Evaluation of the Association Between Gastric Acid Suppression and Risk of Intestinal Colonization With Multidrug-Resistant Microorganisms: A Systematic Review and Meta-analysis

**DOI:** 10.1001/jamainternmed.2020.0009

**Published:** 2020-02-24

**Authors:** Roel P. J. Willems, Karin van Dijk, Johannes C. F. Ket, Christina M. J. E. Vandenbroucke-Grauls

**Affiliations:** 1 Amsterdam Infection and Immunity Institute, Department of Medical Microbiology and Infection Prevention, Amsterdam UMC, Vrije Universiteit Amsterdam, Amsterdam, the Netherlands; 2 Medical Library, Vrije Universiteit Amsterdam, Amsterdam, the Netherlands

## Abstract

**Importance:**

Acid suppressants inhibit gastric acid secretion and disrupt the intestinal microbiome. Whether acid suppression increases the risk of colonization with multidrug-resistant microorganisms (MDROs) is unclear.

**Objectives:**

To systematically examine the association of use of acid suppressants with the risk of colonization with MDROs and to perform a meta-analysis of current evidence.

**Data Sources:**

PubMed, Embase, the Web of Science Core Collection, and the Cochrane Central Register of Controlled Trials were searched from database inception through July 8, 2019.

**Study Selection:**

Study selection was performed independently by 2 authors (R.P.J.W. and C.M.J.E.V.-G.) on the basis of predefined selection criteria; conflicts were resolved by consensus or by an adjudicator (K.v.D.). Human observational studies (case control, cohort, and cross-sectional) and clinical trial designs were selected if they quantified the risk of MDRO colonization in users of acid suppressants in comparison with nonusers.

**Data Extraction and Synthesis:**

The Preferred Reporting Items for Systematic Reviews and Meta-analyses (PRISMA) and Meta-analysis of Observational Studies in Epidemiology (MOOSE) recommendations were followed. Data were extracted independently by the same 2 authors, and adjudication was conducted when necessary. Risk of bias was assessed according to a modified Newcastle-Ottawa Scale. Pooled odds ratios (ORs) were estimated using random-effects models; heterogeneity was evaluated using the *I*^2^ method.

**Main Outcomes and Measures:**

The primary outcome measure was intestinal colonization with MDROs of the Enterobacterales order (producing extended-spectrum β-lactamases, carbapenemases, or plasmid-mediated AmpC β-lactamases), vancomycin-resistant enterococci, methicillin-resistant or vancomycin-resistant *Staphylococcus aureus*, or multidrug-resistant *Pseudomonas* or *Acinetobacter* species.

**Results:**

A total of 26 observational studies including 29 382 patients (11 439 [38.9%] acid suppressant users) met the selection criteria. Primary meta-analysis of 12 studies including 22 305 patients that provided adjusted ORs showed that acid suppression increased the odds of intestinal carriage of MDROs of the Enterobacterales order and of vancomycin-resistant enterococci by roughly 75% (OR = 1.74; 95% CI, 1.40-2.16; *I*^2^ = 68%). The odds were concordant with the secondary pooled analysis of all 26 studies (OR = 1.70; 95% CI, 1.44-1.99; *I*^2^ = 54%). Heterogeneity was partially explained by variations in study setting and the type of acid suppression.

**Conclusions and Relevance:**

Acid suppression is associated with increased odds of MDRO colonization. Notwithstanding the limitations of observational studies, the association is plausible and is strengthened by controlling for confounders. In view of the global increase in antimicrobial resistance, stewardship to reduce unnecessary use of acid suppressants may help to prevent MDRO colonization.

## Key Points

**Question:**

Is gastric acid suppression therapy associated with an increased risk of intestinal colonization with multidrug-resistant microorganisms?

**Findings:**

This systematic review and meta-analysis, including 26 observational studies and 29 382 patients, found that the use of acid suppressants was associated with an increased risk of colonization of the intestinal tract with multidrug-resistant microorganisms of the Enterobacterales order (producing extended-spectrum β-lactamases, carbapenemases, or plasmid-mediated AmpC β-lactamases) and with vancomycin-resistant enterococci.

**Meaning:**

This adverse effect of acid suppressant use adds to others recently described and, in view of the global increase in antimicrobial resistance, calls for a more prudent use of acid suppression therapy, which may help to reduce multidrug-resistant microorganism colonization rates.

## Teaser

This systematic review and meta-analysis examines the association of use of gastric acid suppressants with the risk of colonization with multidrug-resistant microorganisms (MDROs).

## Introduction

Antibiotic resistance is an increasing threat to human health.^1^ Carriers of multidrug-resistant microorganisms (MDROs) are at increased risk for developing infections that are difficult to treat and may contribute to further spread of these strains.^2,3,4,5^ To our knowledge to date, several risk factors for colonization with MDROs have been described, including antibiotic use, age, underlying illness, and international travel.^6,7,8^ Recent evidence has pointed to the use of acid suppression therapy as a possible risk factor for colonization with MDROs.^9,10^

Acid suppressants inhibit stomach acid secretion and can change the composition of the intestinal microbiome^11,12,13^; stomach acid and a healthy intestinal microbiome protect the gastrointestinal tract against colonization by exogenous bacteria.^14^ Whether acid suppression facilitates colonization and infection with MDROs remains unclear. Current evidence from observational studies has been inconsistent, considering that some epidemiologic studies report an increased risk of MDRO colonization with acid suppression,^10^ whereas others do not demonstrate such an association.^15^

During the past couple of decades, acid suppressants have become widely prescribed and are freely available at drugstores.^16^ According to data from the National Health and Nutrition Examination Survey,^17^ nearly 8% of US adults used proton pump inhibitors (PPIs) in 2011 and 2012, a doubling compared with 1999 and 2000. This PPI use is highest in older adults—approximately 17% of those aged 60 to 79 years use PPIs.^18^ In addition, as much as 50% to 70% of PPI use seems to be inappropriate based on incorrect indications or failure to stop use when no longer needed.^19,20,21,22^ In view of the possible risks associated with use of these drugs, we performed a systematic review and meta-analysis to determine whether acid suppression therapy is associated with colonization by MDROs.

## Methods

We followed the Preferred Reporting Items for Systematic Reviews and Meta-analyses (PRISMA) and Meta-analysis of Observational Studies in Epidemiology (MOOSE) reporting guidelines.^23,24^ The protocol was preregistered in PROSPERO (CRD42018092541). In the eMethods in the Supplement, we describe the MDROs eligible for inclusion, search strategies, data collection items, and quality assessment scale in detail.

### Eligibility Criteria

Clinical and observational studies (cohort, case control, and cross-sectional) were selected when they reported the association of acid suppression with the risk of colonization with MDROs in human participants. Eligible studies investigated intestinal carriage with the target MDROs. We considered urinary tract infections (UTIs) to be a proxy of rectal carriage, since most UTIs are caused by bacterial species that colonize the intestinal tract.^25,26^ Therefore, studies of UTI were also included. We placed no restrictions on study setting, size, or location. The inclusion was limited to studies reporting enough data to calculate odds ratios (ORs) and their corresponding 95% CIs. Studies restricted to populations with *Clostridium difficile* were excluded because acid suppression is a well-known risk factor for infection with this microorganism.^27^

### Search Strategy and Study Selection

PubMed, Embase, the Web of Science Core Collection (Clarivate Analytics), and the Cochrane Central Register of Controlled Trials (Wiley-Cochrane Library) were systematically searched from database inception through July 8, 2019 (R.P.J.W. and J.C.F.K.), without language restrictions. We used index terms or free-text words (including synonyms and closely related words) that were associated with MDROs and acid suppressants. Second, we performed a cross-reference check of relevant articles and reviews, supplemented by a search of the European Society of Clinical Microbiology and Infectious Diseases eLibrary. The most up-to-date versions of full-text publications were included.

Study selection was performed in 2 stages using a validated Web application.^28^ First, titles and abstracts were screened; then, selected full-text articles were included according to the eligibility criteria. Screening was performed independently by 2 authors (R.P.J.W. and C.M.J.E.V.-G.). Conflicts were handled by consensus, and an adjudicator (K.v.D.) was consulted when necessary.

### Data Collection

Data were collected independently by R.P.J.W. and C.M.J.E.V.-G. using a predesigned spreadsheet (Excel [Microsoft]) that was pilot-tested beforehand. Conflicts were settled by discussion or adjudication (K.v.D.).

Collected data items included authors, year of publication, study setting and design, participant characteristics, details of acid suppressant use, outcomes, and risk estimators. Acid suppression was categorized according to the Anatomical Therapeutic Chemical classification system.^29^ Most studies defined acid suppressant use as current use or any use within a specific time window before the index date. Corresponding authors were asked via email to clarify or provide additional information.

### Outcomes

The outcome of interest was intestinal colonization with target MDROs. In addition, we included studies investigating the association of UTI with MDROs of the Enterobacterales order (MDR-E).

### Risk of Bias Assessment

Along with data extraction, 2 authors (R.P.J.W. and C.M.J.E.V.-G.) independently judged study quality according to a modified Newcastle-Ottawa Scale^30^ without blinding to authors or journals. Conflicts were resolved either by consensus or by the adjudicator (K.v.D.).

### Statistical Analysis

First, pooled ORs with 95% CIs were estimated using random-effects meta-analysis with the generic inverse-variance method for only studies that provided fully adjusted ORs.^31^ In a second analysis with this same method, we included all studies; in this analysis, fully adjusted ORs were used when available. Inconsistency across studies was measured with the *I*^2^ method. Cutoff values of 25%, 50%, and 75% indicated low, moderate, and high heterogeneity, respectively.^32^ We visualized the results with forest plots.

Second, to examine heterogeneity, we performed analyses of predefined subgroups based on study design and type of acid suppressant studied. Subsequent subgroup analyses were conducted by looking further into target MDROs and study setting. Next, to determine the influence of the surrogate outcome measure of UTI, all analyses were repeated with exclusion of the studies of UTI. Additionally, to address potential bias and verify our results, we performed various sensitivity analyses by (1) excluding low-quality studies, (2) restricting the analysis to high-quality studies that adjusted for classic confounders, (3) using a leave-one-out method, (4) Mantel-Haenszel weighting, and (5) calculating the summary estimate with the Knapp-Hartung modification.^33^

Finally, to investigate the risk of publication bias, we applied the Egger test and the test used by Peters et al^31,34,35^ and visually inspected the funnel plots.All analyses were carried out using Review Manager, version 5.3 (Nordic Cochrane Centre), complemented by STATA statistical software, version 14.1 (StataCorp).

## Results

### Study Selection

**Figure 1.**
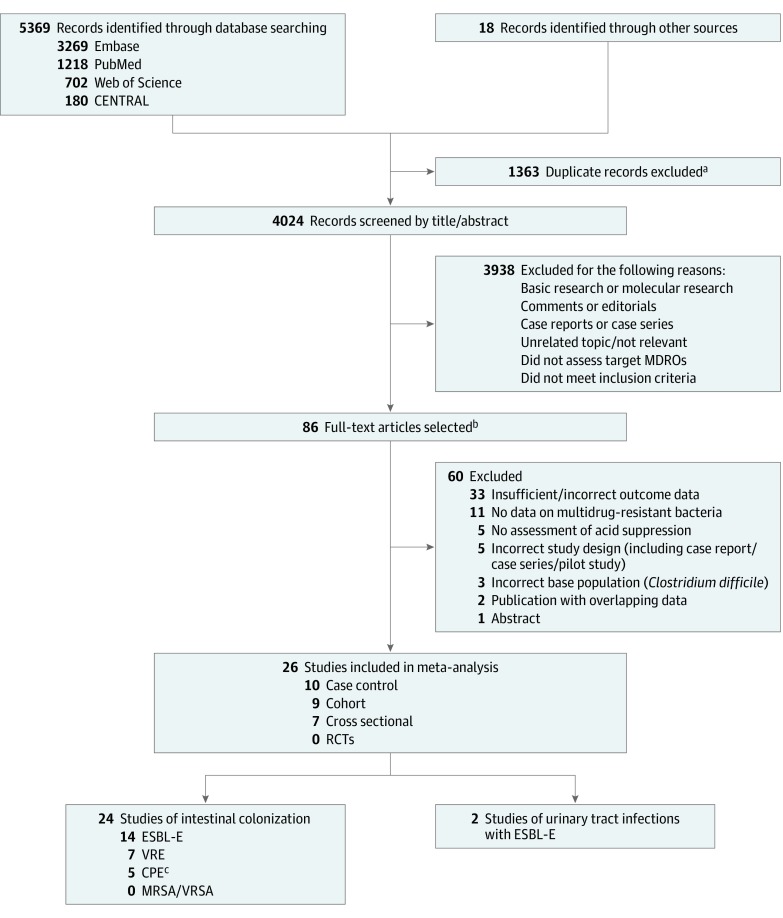
PRISMA Diagram of Study Selection CENTRAL indicates Cochrane Central Register of Controlled Trials; CPE, carbapenemase-producing multidrug-resistant microorganisms of the Enterobacterales order; ESBL-E, extended-spectrum β-lactamase–producing multidrug-resistant microorganisms of the Enterobacterales order; MDROs, multidrug-resistant microorganisms; MRSA, methicillin-resistant *Staphylococcus aureus*; PRISMA, Preferred Reporting Items for Systematic Reviews and Meta-analyses; RCT, randomized clinical trial; VRE, vancomycin-resistant enterococci; VRSA, vancomycin-resistant *S aureus*. ^a^EndNote software (Clarivate Analytics) was used to remove duplicates. ^b^The Cohen κ indicated strong agreement for the full-text stage (κ = 0.82). ^c^Goodman et al^36^ included carbapenemase-producing *Acinetobacter* and *Pseudomonas* species in addition to CPE.

Study selection is presented in Figure 1.^36^ We retained 26 nonduplicate studies that met the purpose of the meta-analysis.^8,9,10,15,36,37,38,39,40,41,42,43,44,45,46,47,48,49,50,51,52,53,54,55,56,57^ Among these 26 studies, 2 clinical studies of interventions not related to the use of acid suppressants were included as cohort studies because no intervention effect was found and they included the analysis of exposure to acid suppressants as a covariate.^50,53^ A total of 24 studies measured intestinal carriage, 19 of MDR-E^8,9,10,15,36,37,39,42,43,44,45,46,48,49,51,52,54,56,57^ and 7 of vancomycin-resistant enterococci (VRE).^38,40,41,47,50,53,55^ Additionally, 2 studies had UTI as the outcome measure.^46,54^ We found no eligible randomized clinical trials and no eligible studies of intestinal colonization with methicillin-resistant *Staphylococcus aureus* or vancomycin-resistant *S aureus.* One study of carbapenemase-producing microorganisms included *Pseudomonas* and *Acinetobacter* species.^36^ We contacted 12 author groups.^8,9,10,37,40,41,42,44,54,55,56,57^ Authors from all but 1 study responded, and those from 5 of the studies provided additional data that we included in the analyses.^8,9,41,55,56^

### Study Characteristics

The 26 studies included 29 382 participants (11 439 [38.9%] were acid suppressant users; 15 866 [54.0%] were female). Twelve studies provided risk estimates that were adjusted for confounding using multivariable analysis.^8,9,10,37,39,40,43,45,47,54,55,57^ Overall, the 12 studies included 22 305 participants (8491 [38.1%] were acid suppressant users; 12 714 [57.0%] were female). Of these, 7 studies were cross-sectional,^9,10,37,43,45,55,57^ 3 were case control,^39,40,54^ and 2 were cohort studies.^8,47^

We summarized the study characteristics in the Table. Studies were published between 1996 and 2019; most were of adult populations (age ≥18 years). Three studies were designed to determine the risk associated with acid suppressants,^9,39,54^ whereas the remaining studies evaluated risk factors in general. Most studies were conducted in the World Health Organization European region (13 of 26 studies) and the region of the Americas (11 of 26 studies) (eTable 1 in the Supplement). Baseline values together with covariates adjusted for, as well as details of exposure and outcome ascertainment, are presented in eTables 2, 3, and 4 in the Supplement.

**Table.** Study Characteristics

Source; Country	Years of Study	Design	Study Setting	Outcome Measure	MDRO Subtype	Acid Suppression Therapy	Sampling Method
**With Statistical Adjustment**							
Arcilla et al,^8^ 2017; the Netherlands	2012-2013	Cohort, prospective, multicenter	Travel clinics	Colonization	ESBL-E	Acid suppression, unspecified	Stool
Ben-Ami et al,^37^ 2006; Israel	2002-2003	Cross-sectional^a^	Tertiary care hospital	Colonization	ESBL-E	PPIs and H_2_RAs	Stool
Cheng et al,^39^ 2016; China	2011-2015	Case control, prospective multicenter^b^	Hospitals (teaching hospital and multiple extended-care hospitals)	Colonization	CPE	PPIs	Stool or rectal swab
Falk et al,^40^ 2000; United States	1996-1997	Case control, retrospective	University hospital burn ICU	Colonization	VRE	Antacids	Rectal swab
Hamprecht et al,^43^ 2016; Germany	2014	Cross-sectional, multicenter^a^	Tertiary care hospitals	Colonization	ESBL-E^c^	Acid suppression, unspecified	Stool or rectal swab
Huizinga et al,^9^ 2017; the Netherlands	2014; 2015	Cross-sectional^a^^,^^b^	Teaching hospital	Colonization	ESBL-E	PPIs and H_2_RAs	Rectal swab
Latour et al,^45^ 2019 Belgium	2015	Cross-sectional, multicenter	Nursing homes	Colonization	ESBL-E	PPIs and H_2_RAs	Rectal swab
McNeil et al,^47^ 2006; United States	2000-2003	Cohort, prospective	Tertiary care hospital liver transplant unit	Colonization	VRE	PPIs	Stool or rectal swab
Reuland et al,^10^ 2016; the Netherlands	2011	Cross-sectional	Community	Colonization	ESBL-E	PPIs, H_2_RAs, and antacids	Stool or perirectal swab
Søgaard et al,^54^ 2017; Denmark	2007-2012	Case control, retrospective^b^	Community	Urinary tract infection	ESBL-E	PPIs	Urine
Tan et al,^55^ 2018; Singapore	2014; 2015; 2016	Cross-sectional, multicenter	Mixed (acute-care hospital and multiple intermediate-term and long-term care facilities)	Colonization	VRE	PPIs, H_2_RAs, and antacids	Stool or rectal swab
Wielders et al,^57^ 2017; the Netherlands	2014-2015	Cross-sectional	Community	Colonization	ESBL-E; AmpC-E	PPIs	Stool
**Without Statistical Adjustment**							
Chanderraj et al,^38^ 2019; United States	2013-2016	Case control, retrospective	Tertiary care hospital (ICU, hemato-oncology unit, and bone marrow transplant unit)	Colonization	VRE	PPIs	Rectal swab
Ford et al,^41^ 2015; United States	2006-2012	Cohort, retrospective	Tertiary care hospital (hematology and bone marrow transplant units)	Colonization	VRE	PPIs	Stool
Goodman et al,^36^ 2019; United States	2016-2017	Cross-sectional^a^	Teaching hospital (medical ICU or solid-organ transplant unit)	Colonization	CPO^d^	PPIs and H_2_RAs	Perirectal swab
Hagel et al,^42^ 2019; Germany	2013-2015	Cohort, prospective	University hospital	Colonization	ESBL-E	Acid suppression, unspecified	Rectal swab
Kuenzli et al,^44^ 2014; Switzerland	2012-2013	Cohort, prospective, multicenter	Travel clinics	Colonization	ESBL-E	PPIs	Rectal swab
Lee et al,^46^ 2018; Republic of Korea	2015-2016	Case control, retrospective	University hospital emergency department	Urinary tract infection	ESBL-E	PPIs	Urine
Okamoto et al,^15^ 2017; United States	2012-2013	Case control, prospective, multicenter	Long-term acute-care hospitals	Colonization	KPC-E	PPIs and H_2_RAs	Rectal swab
Östholm-Balkhed et al,^48^ 2013; Sweden	2008-2009	Cohort, prospective, multicenter	Vaccination clinics	Colonization	ESBL-E	Acid suppression, unspecified	Stool
Prasad et al,^49^ 2016; United States	NA	Cross-sectional	Long-term care facility	Colonization	KPC-E	PPIs	Rectal swab
Puzniak et al,^50^ 2001; United States^e^	1997-1998	Cohort, prospective	Tertiary care hospital medical ICU	Colonization	VRE	Acid suppression, unspecified	Stool or rectal swab
Rodríguez-Baño et al,^51^ 2008; Spain	2005-2006	Cross-sectional	Community	Colonization	ESBL-E	Acid suppression, unspecified	Stool
Seekatz et al,^52^ 2018; United States	2014-2016	Case control, prospective	Long-term acute-care hospital	Colonization	KPC-E	PPIs	Stool or rectal swab
Slaughter et al,^53^ 1996; United States^e^	1994-1995	Cohort, prospective	Teaching hospital medical ICU	Colonization	VRE	PPIs, H_2_RAs, and antacids	Rectal swab
Vading et al,^56^ 2016; Sweden	2013-2015	Cohort, prospective	Travel clinic	Colonization	ESBL-E	PPIs and antacids	Rectal swab

Abbreviations: AmpC-E, plasmid-mediated AmpC β-lactamase–producing multidrug-resistant microorganisms of the Enterobacterales order; CPE, carbapenemase-producing multidrug-resistant microorganisms of the Enterobacterales order; CPO, carbapenemase-producing organisms; ESBL-E, extended-spectrum β-lactamase–producing multidrug-resistant microorganisms of the Enterobacterales order; H_2_RA, histamine_2_ receptor antagonist; ICU, intensive care unit; KPC-E, *Klebsiella pneumoniae* carbapenemase-producing multidrug-resistant microorganisms of the Enterobacterales order; MDROs, multidrug-resistant microorganisms; NA, not available; PPI, proton pump inhibitor; VRE, vancomycin-resistant enterococci.

^a^ Studies that used screening at admission to the hospital.

^b^ Studies specifically designed to assess the risk associated with acid suppression (all other studies evaluated acid suppression as 1 risk factor among many).

^c^ Study assessed third-generation cephalosporin-resistant MDR-E; ESBL was the predominant resistance mechanism detected in 90% of the isolates.

^d^ Study assessed carbapenemase-producing glucose-nonfermenting Gram-negative MDR-E in addition to CPE.

^e^ Intervention studies analyzed as cohort studies.

### Risk of Bias and Primary Analysis

**Figure 2.**
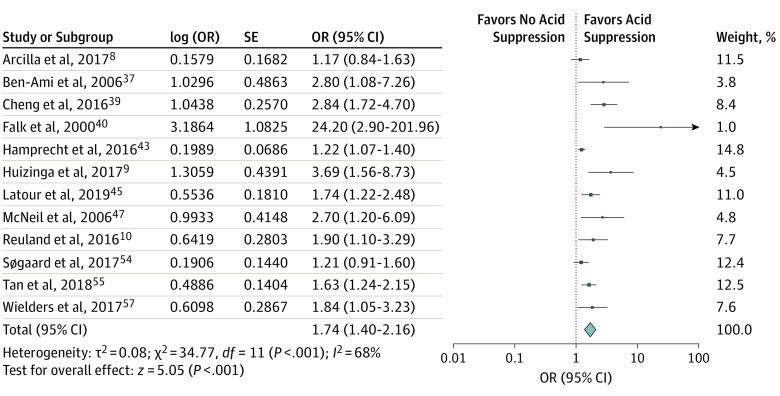
Forest Plot for the Association of Multidrug-Resistant Microorganism Colonization With Acid Suppression Odds ratios (ORs) are presented as random effects with inverse variance (except for the log [OR] column). Among studies, acid suppression mainly included exposure to proton pump inhibitors and/or histamine_2_ receptor antagonists, with few studies including other antacids.

The median (range) Newcastle-Ottowa Scale^30^ score was 6 (3-9) (eTable 5 in the Supplement). In the primary analysis, we included the 12 studies that adjusted for confounding.^8,9,10,37,39,40,43,45,47,54,55,57^ This showed that acid suppression was associated with MDRO colonization (OR = 1.74; 95% CI, 1.40-2.16) (Figure 2). Among these studies, heterogeneity, as measured using the *I*^2^ method, was 68%. Restriction of the analysis to the 11 studies^8,9,10,37,39,40,43,45,47,55,57^ that directly evaluated intestinal carriage (not UTI) yielded a summary OR of 1.86 (95% CI, 1.46-2.37); heterogeneity remained the same (*I*^2^ = 70%).

### Secondary Analysis

A secondary analysis of all 26 studies revealed odds consistent with those found in the primary analysis and showed that acid suppression was associated with MDRO colonization (OR = 1.70; 95% CI, 1.44-1.99; *I*^2^ = 54%) (eFigure 1 in the Supplement). Analysis of the 24 studies^8,9,10,15,36,37,38,39,40,41,42,43,44,45,47,48,49,50,51,52,53,55,56,57^ that directly evaluated intestinal carriage yielded an OR of 1.77 (95% CI, 1.48-2.10; *I*^2^ = 56%).

### Subgroup Analysis

#### By MDRO Subtype

**Figure 3.**
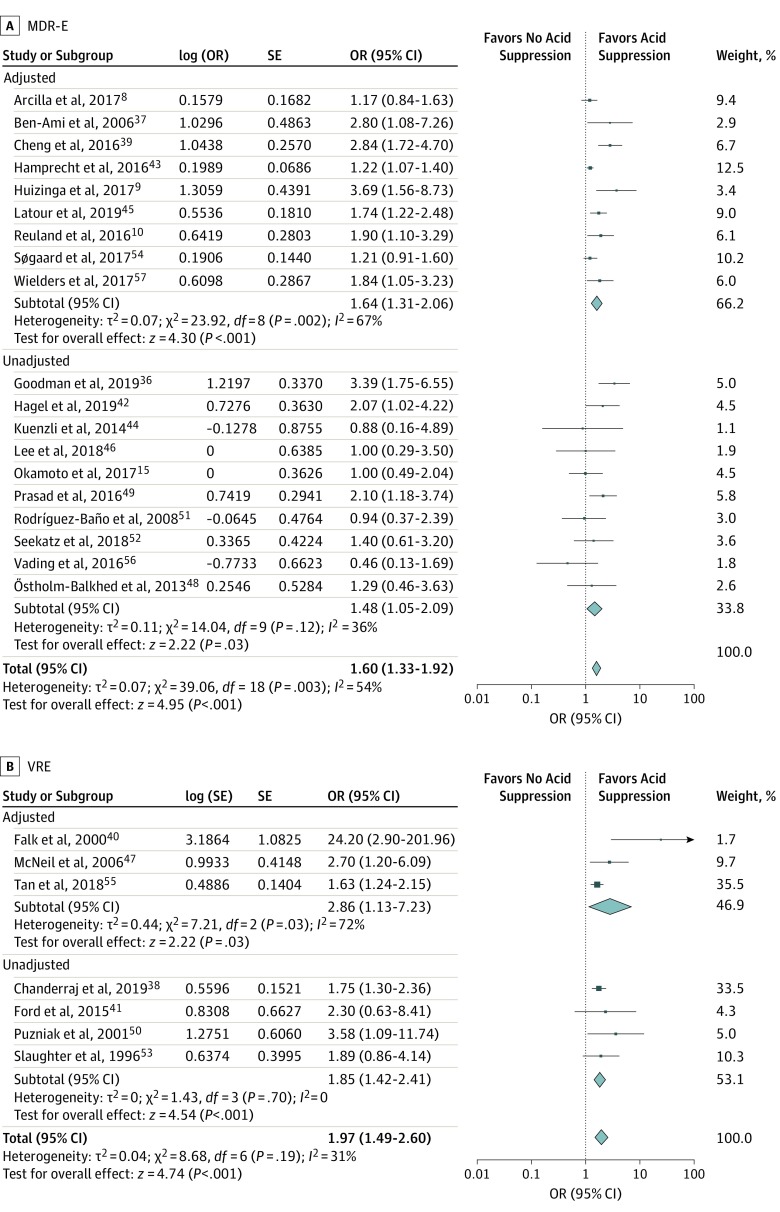
Subgroup Analysis by Multidrug-Resistant Microorganism Subtype A, Multidrug-resistant microorganisms of the Enterobacterales order (MDR-E). B, Vancomycin-resistant enterococci (VRE). Odds ratios (ORs) are presented as random effects with inverse variance (except for the log [OR] column).

**Figure 4.**
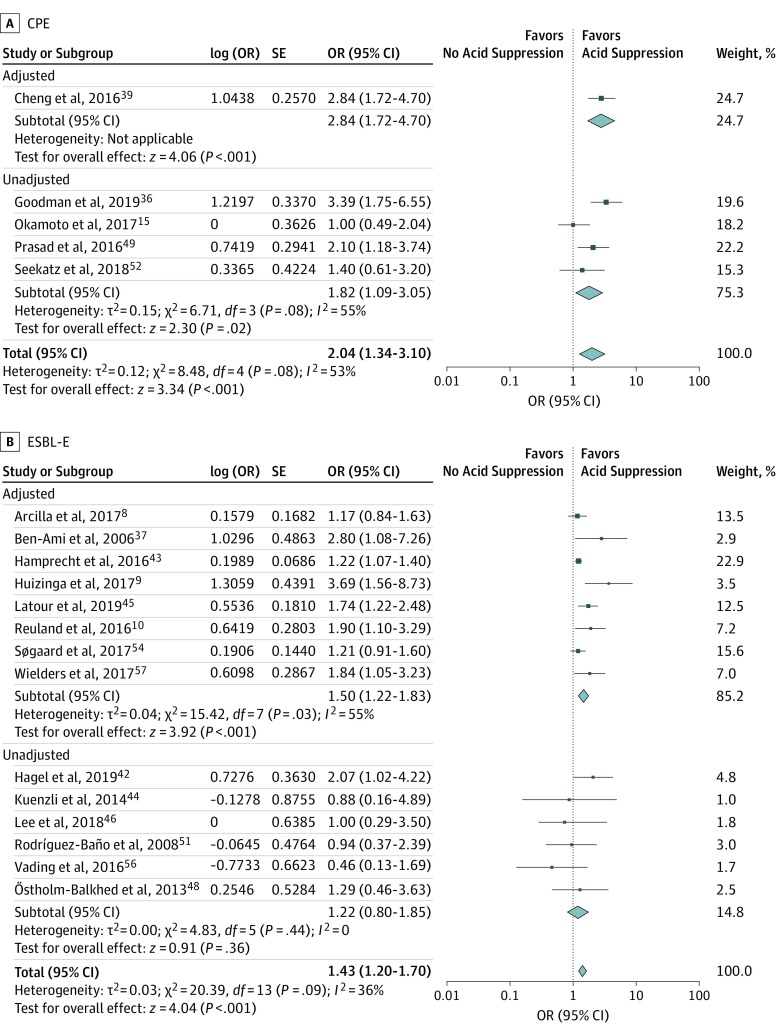
Subgroup Analysis by Multidrug-Resistant Microorganism Subtype A, Carbapenemase-producing multidrug-resistant microorganisms of the Enterobacterales order (CPE). B, Extended-spectrum β-lactamase–producing multidrug-resistant microorganisms of the Enterobacterales order (ESBL-E). Odds ratios (ORs) are presented as random effects with inverse variance (except for the log [OR] column).

Acid suppression was associated with MDR-E carriage as well as VRE carriage (MDR-E: OR = 1.60; 95% CI, 1.33-1.92; *I*^2^ = 54%; VRE: OR = 1.97; 95% CI, 1.49-2.60; *I*^2^ = 31%). The association was larger for carbapenemase-producing MDR-E (CPE) than for extended-spectrum β-lactamase–producing MDR-E (ESBL-E), although the ORs had overlapping CIs (CPE: OR = 2.04; 95% CI, 1.34-3.10; *I*^2^ = 53%; ESBL-E: OR = 1.43; 95% CI, 1.20-1.70; *I*^2^ = 36%) (Figure 3 and Figure 4).

#### By Design

To evaluate the influence of research methods, we performed a subgroup analysis by study design. Overall, the association of acid suppression therapy with MDRO colonization was marginally moderated by study design (cohort: OR = 2.31; 95% CI, 1.56-3.43; *I*^2^ = 0%; case control: OR = 1.64; 95% CI, 1.13-2.38; *I*^2^ = 66%; cross-sectional: OR = 1.84; 95% CI, 1.47-2.30; *I*^2^ = 58%) (eFigure 2 in the Supplement).

#### By Type of Acid Suppressant

To evaluate whether the association depended on the type of acid suppressant used, we restricted the analysis to PPI users because PPIs exert more potent acid suppression than histamine_2_ receptor antagonists (H_2_RAs).^58^ Seventeen studies^9,10,36,37,38,39,41,44,45,46,47,49,52,54,55,56,57^ reported the risk of MDRO colonization in PPI users only; the meta-analysis yielded an OR of 1.81 (95% CI, 1.52-2.16; *I*^2^ = 33%). Four studies^9,10,37,55^ reported risk in H_2_RA users only. Use of these drugs did not seem to be associated with MDRO colonization (OR = 1.33; 95% CI, 0.86-2.08; *I*^2^ = 15%) (eFigure 3 in the Supplement); the large CI suggests that the lack of association may be due to the small number of studies.

#### By Setting

We divided the 15 hospital-based studies into 2 groups: 4 studies^9,36,37,43^ evaluated colonization at admission (screening within 48 hours of admission), and 11 studies^15,38,39,40,41,42,46,47,50,52,53^ evaluated colonization during hospital stay. The OR of colonization with MDROs at hospital admission was 2.39 (95% CI, 1.17-4.87; *I*^2^ = 82%). Meta-analysis of the studies that focused on colonization during hospital stay showed a similar association (OR = 1.98; 95% CI, 1.50-2.62; *I*^2^ = 33%); 4 community-based studies^10,51,54,57^showed similar results but with a slightly smaller association (OR = 1.41; 95% CI, 1.07-1.87; *I*^2^ = 21%). However, meta-analysis of 4 travel-based studies^8,44,48,56^ yielded an OR with a very broad CI (OR = 1.11; 95% CI, 0.82-1.50; *I*^2^ = 0%) (eFigure 4 in the Supplement). Three studies were conducted in residents of long-term care facilities and were therefore excluded from this subgroup analysis.^45,49,55^

### Sensitivity Analysis

To ascertain the strength of our results, we performed additional sensitivity analyses (eTables 6, 7, and 8 and eFigures 5 and 6 in the Supplement). The results were consistent; the association remained significant in all analyses.

Both Mantel-Haenszel weighting and the Knapp-Hartung^33^ estimators yielded similar results. Using the leave-one-out method, we found no studies that influenced the results disproportionately (lowest value: OR = 1.64; 95% CI, 1.40-1.92; highest value: OR = 1.75; 95% CI, 1.49-2.07).

Restriction of the analyses to high-quality studies of intestinal carriage did not substantially change the summary estimate (OR = 1.74; 95% CI, 1.42-2.14; *I*^2^ = 64%).^8,9,10,15,36,38,39,43,50,52,53,55,57^ Four of these studies adjusted for at least age, sex, and antibiotic use and had a maximum Newcastle-Ottawa Scale^30^ score for ascertainment of the exposure; their summary estimate (OR = 2.15; 95% CI, 1.52-3.04; *I*^2^ = 49%)^9,10,39,55^ was similar to that of the primary meta-analysis.

### Publication Bias

We observed no evidence of publication bias with inspection of the funnel plot or with the Egger test or the test used by Peters et al.^31,34,35^ Excluding both studies of UTI did not affect publication bias estimators (eFigure 7 in the Supplement).

## Discussion

This systematic review and meta-analysis showed that the use of acid suppressants (mainly PPIs or H_2_RAs) is associated with a 75% increase in the odds of intestinal MDRO colonization, both in the community and in the health care setting. This association was found in a primary analysis of the 12 studies^8,9,10,37,39,40,43,45,47,54,55,57^ covering more than 22 000 patients, which provided adjusted risk estimates, as well as in the secondary analysis of all studies^8,9,10,15,37,38^ (>29 000 patients). The risk was similar for colonization with Gram-negative MDR-E and Gram-positive enterococci. The results from our sensitivity analyses, in which we address the risk of bias and confounding, buttress these findings.

Acid suppressants may promote colonization with MDROs through 3 different mechanisms. First, and most important, acid suppressants reduce gastric acid secretion; this is associated with bacterial survival and in turn the amount of viable exogenous bacteria that pass through the stomach to reach the intestine.^59^ Second, such agents have been shown to directly alter the composition of intestinal microbiota, leading to a decrease in mean species diversity.^11,12,13^ This may influence microbiota-mediated colonization resistance. For bacterial species such as VRE and MDR-E, resistance to colonization can be induced by microbiota-driven immune responses or by targeted depletion of nutrients or toxic substances.^14^ Third, a 2019 study of ESBL-producing *Escherichia coli* sequence type 131^60^ showed that these strains contain several protein amino acid substitutions that confer resistance to gastric acid. Therefore, MDROs might be better able to pass the gastric acid barrier. This characteristic may present an additional advantage, even in a gastric environment where this barrier is less effective than normal as a consequence of acid suppressant use.

Acid suppression conferred the largest risk for colonization with VRE and CPE (nearly 2-fold higher odds), whereas for ESBL-E, the OR was approximately 1.4. However, these differences should be interpreted with caution because the CIs of the ORs overlap.

We explored the association according to type of study design and setting. These did not influence the estimates substantially; the odds of MDRO colonization with acid suppression therapy remained nearly 2-fold higher. An exception was found for PPI use among travelers; in this group, there was not an association. However, it is conceivable that the small proportion of acid suppressant users in the traveler cohorts (between 3% and 12% of the total cohort) precluded the identification of an association. In addition, the influence of individual risk factors on the acquisition of intestinal carriage may be overshadowed by the large risk posed by travel to endemic regions.^44^ Up to 75% of travelers to southern Asia return with ESBL-E in their stool.^8^

Since the acid suppression induced by PPIs is more profound than that caused by H_2_RAs, we expected the association of PPI use with MDRO colonization to be larger than that of H_2_RA with MDRO colonization.^58^ The risk associated with PPI use was larger than the risk associated with H_2_RAs. However, the number of studies of H_2_RAs was small (n = 4),^9,10,37,55^ and the CI of the estimate was large. Therefore, to clearly define a difference in the associations of PPIs and H_2_RAs with MDRO colonization, more studies of H_2_RAs are needed.

Unfortunately, only 2 of the studies reported dose or duration of acid suppression therapy. These 2 studies, both of VRE colonization, did find an association of duration of acid suppressant exposure with increased risk of VRE colonization.^38,40^

### Strengths

To the best of our knowledge, this is the first systematic review and meta-analysis to date of the association of gastric acid suppression with MDRO colonization. We were able to include 12 studies^8,9,10,37,39,40,43,45,47,54,55,57^ with adjusted ORs, comprising more than 22 000 patients; this large sample yielded an accurate estimate of the effect size. Inclusion of the studies that did not provide adjusted ORs in the analysis yielded the same results. We incorporated several sensitivity analyses to test whether our findings were robust. A major strength is that we strictly adhered to the PRISMA and MOOSE guidelines, following a focused hypothesis.^23,24^ We applied stringent criteria and restricted our review to studies that analyzed the presence of MDROs in the gastrointestinal tract, the site of action of acid suppression therapy, and the main route of acquisition of the MDROs (ie, MDR-E or enterococci).

### Limitations

Our study has some limitations. The studies included in the analysis were heterogeneous, partly owing to differences in exposure and study setting. Nevertheless, we believe the effect of heterogeneity to be small given the steady summary estimates across the subgroup and sensitivity analyses.

This meta-analysis is based on observational studies, which are potentially limited by confounding factors such as age, sex, comorbidity, and especially antibiotic use. Users of PPIs may differ in lifestyle and severity of disease (possibly causing confounding by disease severity). However, analysis of the studies that adjusted for potential confounders showed that the odds of colonization with MDROs were consistently increased by use of acid suppressants.^8,9,10,37,39,40,43,45,47,54,55,57^ Furthermore, the adjusted group estimates were higher overall than the unadjusted group estimates across all analyses performed.

We included only 2 studies^46,54^ that investigated the surrogate outcome measure of UTI. Therefore, we cannot draw conclusions about whether the use of acid suppressants also increases the risk of infection with MDROs, irrespective of the association with intestinal carriage. However, the current literature underpins the concept of the intestinal reservoir; intestinal colonization appears to be an important intermediary step toward infection.^2,3,4,5^

## Conclusions

In conclusion, our systematic review showed that acid suppression is associated with an increased risk of colonization with MDROs. This association is biologically plausible but should be interpreted with caution, since evidence from observational studies cannot prove causation. However, this adverse effect adds to many others that were described recently, such as the increased risks of *Clostridium difficile* colitis, bacterial gastroenteritis, and renal diseases.^27,61,62,63,64^ We advocate that acid suppressants should be used when necessary but that unnecessary use should be avoided.

Because up to 70% of PPI prescriptions appear to be based on indications without clear benefit^20,21^ and in view of the ever-growing problem of antimicrobial resistance, we see the possibility of a favorable interaction between infection control, antibiotic stewardship, and the promotion of rational use of PPIs. This rational use could be called PPI stewardship. Future intervention programs may provide further insight about whether the risks of MDRO colonization and infection are reduced after discontinuation of inappropriate acid suppression therapy.

## Notes

Supplement. **eMethods.** Supplemental Methods. **eTable 1.** Study Distribution According to World Health Organization Region. **eTable 2.** Baseline Characteristics. **eTable 3.** Exposure and Outcome Ascertainment. **eTable 4.** Adjusted Study Results. **eTable 5.** Quality Assessment According to the Modified Newcastle-Ottawa Scale. **eTable 6.** Sensitivity Analyses After Exclusion of Studies on Urinary Tract Infections. **eTable 7.** Sensitivity Analysis by the Leaving-One-Out Method. **eTable 8.** Sensitivity Analysis by Knapp-Hartung Modification. **eFigure 1.** Secondary Analysis. **eFigure 2.** Subgroup Analysis by Design. **eFigure 3.** Subgroup Analysis by Type of Acid Suppressant. **eFigure 4.** Subgroup Analysis by Setting. **eFigure 5.** Sensitivity Analysis of Studies With High Quality. **eFigure 6.** Sensitivity Analyses by Mantel-Haenszel Weighting. **eFigure 7.** Funnel Plot. **eReferences.**
