# Supplementary material for: Evaluation of the Association Between Gastric Acid Suppression and Risk of Intestinal Colonization With Multidrug-Resistant Microorganisms: A Systematic Review and Meta-analysis
Source: JAMA Intern Med. 2020 Feb 24;180(4):561–71. doi: 10.1001/jamainternmed.2020.0009 (PMC7042870; doi:10.1001/jamainternmed.2020.0009)
Supplement: Supplement. — eMethods. Supplemental Methods. eTable 1. Study Distribution According to World Health Organization Region. eTable 2. Baseline Characteristics. eTable 3. Exposure and Outcome Ascertainment. eTable 4. Adjusted Study Results. eTable 5. Quality Assessment According to the Modified Newcastle-Ottawa Scale. eTable 6. Sensitivity Analyses After Exclusion of Studies on Urinary Tract Infections. eTable 7. Sensitivity Analysis by the Leaving-One-Out Method. eTable 8. Sensitivity Analysis by Knapp-Hartung Modification. eFigure 1. Secondary Analysis. eFigure 2. Subgroup Analysis by Design. eFigure 3. Subgroup Analysis by Type of Acid Suppressant. eFigure 4. Subgroup Analysis by Setting. eFigure 5. Sensitivity Analysis of Studies With High Quality. eFigure 6. Sensitivity Analyses by Mantel-Haenszel Weighting. eFigure 7. Funnel Plot. eReferences. [file jamainternmed-180-561-s001.pdf]

Supplementary Online Content

Willems RPJ, van Dijk K, Ket JCF, Vandenbroucke-Grauls CMJE. Evaluation of the association between gastric acid suppression and risk of intestinal colonization with multidrug-resistant microorganisms: a systematic review and meta-analysis. Published online February 24, 2020. *JAMA Intern Med*. doi:10.1001/jamainternmed.2020.0009

eMethods. Supplemental Methods

eTable 1. Study Distribution According to World Health Organization Region

eTable 2. Baseline Characteristics

eTable 3. Exposure and Outcome Ascertainment

eTable 4. Adjusted Study Results

eTable 5. Quality Assessment According to the Modified Newcastle-Ottawa Scale

eTable 6. Sensitivity Analyses After Exclusion of Studies on Urinary Tract Infections

eTable 7. Sensitivity Analysis by the Leaving-One-Out Method

eTable 8. Sensitivity Analysis by Knapp-Hartung Modification

eFigure 1. Secondary Analysis

eFigure 2. Subgroup Analysis by Design

eFigure 3. Subgroup Analysis by Type of Acid Suppressant

eFigure 4. Subgroup Analysis by Setting

eFigure 5. Sensitivity Analysis of Studies With High Quality

eFigure 6. Sensitivity Analyses by Mantel-Haenszel Weighting

eFigure 7. Funnel Plot

eReferences.

This supplementary material has been provided by the authors to give readers additional information about their work.

eMethods. Supplemental Methods
Target Multidrug-Resistant Micro-organisms:

The following drug-resistant pathogens were eligible for inclusion: (1) plasmid-mediated AmpC-β-lactamase-producing or extended-spectrum β-lactamase-producing Enterobacterales (ESBL-E); (2) carbapenemase-producing Enterobacterales (CPE) (ESBL-E and CPE were collectively called multidrug-resistant Enterobacterales, MDR-E); (3) Methicillin-resistant *Staphylococcus aureus* (MRSA); (4) vancomycin-resistant *Staphylococcus aureus* (VRSA); (5) vancomycin-resistant *Enterococcus* (VRE); (6) carbapenem-resistant *Pseudomonas* species (e.g. *Pseudomonas aeruginosa*) and (7) carbapenem-resistant *Acinetobacter* species (e.g. *Acinetobacter baumannii*).

© 2020 Willems RPJ et al. *JAMA Intern Med.*

Search Strategies:

Search strategy for PubMed (July 8, 2019)

#	Searches	Results
#1	"Proton Pump Inhibitors"[Mesh] OR "Proton Pump Inhibitors"[Pharmacological Action] OR proton pump inhibitor*[tiab] OR ppi[tiab] OR ppis[tiab] OR omeprazol*[tiab] OR esomeprazol*[tiab] OR pantoprazol*[tiab] OR lansoprazol*[tiab] OR dexlansoprazol*[tiab] OR rabeprazol*[tiab] OR "Histamine H2 Antagonists"[Mesh] OR "Histamine H2 Antagonists"[Pharmacological Action] OR h2 antagonist*[tiab] OR h2 block*[tiab] OR ranitidin*[tiab] OR cimetidin*[tiab] OR famotidin*[tiab] OR nizatidin*[tiab] OR "Antacids"[Mesh] OR "Antacids"[Pharmacological Action] OR alkalinizing agent*[tiab] OR antacid*[tiab] OR "Anti-Ulcer Agents"[Mesh] OR "Anti-Ulcer Agents"[Pharmacological Action] OR acid suppress*[tiab] OR acid inhibit*[tiab] OR ((h2 receptor*[tiab] OR histamine 2*[tiab] OR "Receptors, Histamine H2"[Mesh]) AND (antagonist*[tiab] OR block*[tiab])) OR h2ra*[tiab] OR anti acid*[tiab] OR anti ulcer*[tiab] OR anti secretory*[tiab] OR antisecretory*[tiab]	109,415
#2	"Drug Resistance, Bacterial"[Mesh] OR "beta-Lactam Resistance"[Mesh] OR "beta-Lactamases"[Mesh] OR esbl*[tiab] OR ampc[tiab] OR ((drug[tiab] OR beta lactam*[tiab] OR "carbapenemase" [Supplementary Concept] OR "beta-Lactams"[Mesh] OR carbapenem*[tiab]) AND resist*[tiab] AND bacteria*[tiab]) OR carbenicillinase*[tiab] OR cephalexin amidase*[tiab] OR cephalosporinas*[tiab] OR exopenicillinas*[tiab] OR penicillinas*[tiab] OR "Genes, MDR"[Mesh] OR multidrug efflux pump gene*[tiab] OR multidrug resist*[tiab] OR multi drug resist*[tiab] OR multiple drug resist*[tiab] OR pampc[tiab] OR metallo beta lactamase*[tiab] OR extended spectrum cephalosporin*[tiab] OR expanded spectrum cephalosporin*[tiab] OR methicillin*[tiab] OR vancomycin*[tiab] OR vre*[tiab] OR mrsa*[tiab] OR vrsa*[tiab] OR visa*[tiab] OR mdr*[tiab]	199,138
#3	#1 AND #2	1,218

Search strategy for Embase.com (July 8, 2019)

#	Searches	Results
#1	'proton pump inhibitor'/exp OR 'histamine h2 receptor antagonist'/exp OR 'antiulcer agent'/exp OR 'proton pump inhibitor*':ti,ab,kw OR ppi:ti,ab,kw OR ppis:ti,ab,kw OR omeprazol*:ti,ab,kw OR esomeprazol*:ti,ab,kw OR pantoprazol*:ti,ab,kw OR lansoprazol*:ti,ab,kw OR dexlansoprazol*:ti,ab,kw OR rabeprazol*:ti,ab,kw OR 'h2 antagonist*':ti,ab,kw OR 'h2 block*':ti,ab,kw OR ranitidin*:ti,ab,kw OR cimetidin*:ti,ab,kw OR famotidin*:ti,ab,kw OR nizatidin*:ti,ab,kw OR 'alkalinizing agent*':ti,ab,kw OR antacid*:ti,ab,kw OR 'acid suppress*':ti,ab,kw OR 'acid inhibit*':ti,ab,kw OR h2ra*:ti,ab,kw OR 'anti acid*':ti,ab,kw OR 'anti ulcer*':ti,ab,kw OR 'anti secretory*':ti,ab,kw OR antisecretory*:ti,ab,kw OR (('h2 receptor*':ti,ab,kw OR 'histamine 2':ti,ab,kw OR 'histamine 2r':ti,ab,kw OR 'histamine h2 receptor'/exp) AND (antagonist*:ti,ab,kw OR block*:ti,ab,kw))	291,041
#2	'antibiotic resistance'/exp OR 'beta lactamase'/exp OR 'multidrug resistance'/exp OR esbl* OR ampc OR ((drug:ti,ab,kw OR 'beta lactam*':ti,ab,kw OR 'carbapenemase'/exp OR 'beta lactam'/exp OR carbapenem*:ti,ab,kw) AND resist*:ti,ab,kw AND bacteria*:ti,ab,kw) OR carbenicillinase*:ti,ab,kw OR 'cephalexin amidase*':ti,ab,kw OR 'cephalosporinas*':ti,ab,kw OR exopenicillinas*:ti,ab,kw OR penicillinas*:ti,ab,kw OR 'multidrug efflux pump gene*':ti,ab,kw OR 'multidrug resist*':ti,ab,kw OR 'multi drug resist*':ti,ab,kw OR 'multiple drug resist*':ti,ab,kw OR pampc:ti,ab,kw OR 'metallo beta lactamase*':ti,ab,kw OR 'extended spectrum cephalosporin*':ti,ab,kw OR 'expanded spectrum	32,3977

© 2020 Willems RPJ et al. *JAMA Intern Med.*

	cephalosporin*':ti,ab,kw OR methicillin*:ti,ab,kw OR vancomycin*:ti,ab,kw OR vre*:ti,ab,kw OR mrsa*:ti,ab,kw OR vrsa*:ti,ab,kw OR visa*:ti,ab,kw OR mdr*:ti,ab,kw	
#3	#1 AND #2	4,443
#4	#3 NOT ('conference abstract'/it OR 'conference paper'/it OR 'conference review'/it OR 'editorial'/it OR 'letter'/it OR 'note'/it)	3,269

Search strategy for Clarivate Analytics Web of Science Core Collection (July 8, 2019)

Indexes=SCI-EXPANDED, SSCI, A&HCI, ESCI Timespan=All years

#	Searches	Results
#1	**TOPIC:** ("proton pump inhibitor*" OR ppi OR ppis OR omeprazol* OR esomeprazol* OR pantoprazol* OR lansoprazol* OR dexlansoprazol* OR rabeprazol* OR "h2 antagonist*" OR "h2 block*" OR ranitidin* OR cimetidin* OR famotidin* OR nizatidin* OR "alkalinizing agent*" OR antacid* OR "acid suppress*" OR "acid inhibit*" OR h2ra* OR "anti acid*" OR "anti ulcer*" OR "anti secretory*" OR antisecretory* OR (("h2 receptor*" OR "histamine 2" OR "histamine 2r") NEAR/3 (antagonist* OR block*)))	75,976
#2	**TOPIC:** (esbl* OR ampc OR ((drug OR "beta lactam*" OR carbapenem*) NEAR/3 resist* NEAR/3 bacteria*) OR carbenicillinase* OR "cephalexin amidase*" OR "cephalosporinas*" OR exopenicillinas* OR penicillinas* OR "multidrug efflux pump gene*" OR "multidrug resist*" OR "multi drug resist*" OR "multiple drug resist*" OR pampc OR "metallo beta lactamase*" OR "extended spectrum cephalosporin*" OR "expanded spectrum cephalosporin*" OR methicillin* OR vancomycin* OR vre* OR mrsa* OR vrsa* OR visa* OR mdr*)	164,639
#3	#1 AND #2	702

Search strategy for Wiley Cochrane Library – CENTRAL (July 8, 2019)

#	Searches	Results
#1	("proton pump inhibitor*" OR ppi OR ppis OR omeprazol* OR esomeprazol* OR pantoprazol* OR lansoprazol* OR dexlansoprazol* OR rabeprazol* OR "h2 antagonist*" OR "h2 block*" OR ranitidin* OR cimetidin* OR famotidin* OR nizatidin* OR "alkalinizing agent*" OR antacid* OR "acid suppress*" OR "acid inhibit*" OR h2ra* OR "anti acid*" OR "anti ulcer*" OR "anti secretory*" OR antisecretory* OR (("h2 receptor*" OR "histamine 2" OR "histamine 2r") NEAR/3 (antagonist* OR block*))):ti,ab,kw	16,153
#2	(esbl* OR ampc OR ((drug OR "beta lactam*" OR carbapenem*) NEAR/3 resist* NEAR/3 bacteria*) OR carbenicillinase* OR "cephalexin amidase*" OR "cephalosporinas*" OR exopenicillinas* OR penicillinas* OR "multidrug efflux pump gene*" OR "multidrug resist*" OR "multi drug resist*" OR "multiple drug resist*" OR pampc OR "metallo beta lactamase*" OR "extended spectrum cephalosporin*" OR "expanded spectrum cephalosporin*" OR methicillin* OR vancomycin* OR vre* OR mrsa* OR vrsa* OR visa* OR mdr*):ti,ab,kw	7,484
#3	#1 AND #2	188
		Records in CENTRAL: 180

© 2020 Willems RPJ et al. *JAMA Intern Med.*

Data Items:

General study characteristics
Primary author and year of publication, time period of study, location (country, WHO region), study setting and design.

Participant characteristics
Sample size, inclusion and exclusion criteria, baseline values for age, sex, overall comorbidity, ethnicity/race, and prior antibiotic exposure.

Exposure
Definition of exposure, including type of acid suppressant (PPIs, H2RAs, and/or antacids), method of ascertainment of the exposure (medical records, questionnaires, information databases); dose, duration and frequency of use, if possible.

Outcome
Definition of outcome, including target micro-organism and resistance mechanism, method of ascertainment of the outcome, including detection (screening) method, method for antibiotic susceptibility testing with interpretative guidelines used, and method of MDRO characterization (phenotypic or genotypic characterization), sampling method (stools, rectal swabs, perirectal swabs).

Outcome measurement details
Raw numbers (no. of events and no. of non-events, no. of exposed and no. of non-exposed participants), (maximally) adjusted odds ratios (and 95% CIs) including the covariates adjusted for.

© 2020 Willems RPJ et al. *JAMA Intern Med.*

Modified Newcastle-Ottawa Scale:

Depending on study quality, stars were awarded in three main categories: (1) selection of groups, (2) comparability of groups, and (3) ascertainment of either exposure or outcome of interest.

Assessment scale for case-control studies:

Selection

Allocate one star for each numbered item (maximum of 4 stars within this category):

1. Adequate case definition with independent validation ①
2. Consecutive or obviously representative series of cases ①
3. Community controls ①
4. Controls with no history of disease (endpoint) ①

Comparability

Allocate one star for each numbered item (maximum of 2 stars within this category):

1. Cases and controls matched in the design and/or adjusted for age and sex in the analysis ①
2. Controlled for antibiotic use or comorbidity ①

Exposure

Allocate one star for each numbered item (maximum of 4 stars within this category) [for item 1, either 1 or 2 stars can be allocated]

1. Ascertainment of exposure with (a) secure records ② (2 stars) or (b) structured interviews (blinded fashion) or structured questionnaires ① (1 star)
2. Same method of ascertainment for cases and controls ①
3. Same non-response rate for both groups ①

Assessment scale for cohort and cross-sectional studies:

Selection

Allocate one star for each numbered item (maximum of 4 stars within this category) [for item 3, either 1 or 2 stars can be allocated]

1. Exposed cohort is truly or somewhat representative of the average in the community ①
2. Non-exposed cohort drawn from same community as exposed cohort ①
3. Ascertainment of exposure with (a) secure records ② (2 stars) or (b) structured interviews or structured questionnaires ① (1 star)
4. Outcome of interest not present at baseline ①

Comparability

Allocate one star for each numbered item (maximum of 2 stars within this category)

1. Exposed and non-exposed individuals matched in design and/or adjusted for age and sex in the analysis ①
2. Controlled for antibiotic use or comorbidity ①

Outcome

Allocate one star for each numbered item (maximum of 4 stars within this category)

1. Assessment of outcome with independent or blind assessment, or confirmation of the outcome by reference to secure records, or record linkage ①
2. Sufficient follow-up duration ①
3. Complete follow-up or loss to follow-up (less than 20%) unlikely to introduce bias ①

Scoring and rationale:

First, we did not score follow-up duration in any of the included cohort studies, because there is a lack of evidence regarding a sufficient timeframe to observe acquisition. In addition, an adequate follow-up duration may vary across different risk factors. Second, since exposure and outcome are measured simultaneously, we did not score selection item 4 and outcome item 2 and 3 for cross-sectional studies. Third, we judged secure records to better address exposure ascertainment compared to the risk of recall bias associated with questionnaires. As we expected to encounter several studies using questionnaires (encompassing different time windows of exposure), we modified the scale to add more weight to studies that used secure records or medication verification.

© 2020 Willems RPJ et al. *JAMA Intern Med.*

eTable 1. Study Distribution According to World Health Organization Region

Region[a]	No. of studies	No. of participants (no. of events)
European Region[b]	13	17227 (2125)
Region of the Americas	10	6148 (1120)
Western Pacific Region	3	6007 (673)

[a] No studies originate within the African Region, Eastern Mediterranean Region or South-East Asia Region.
[b] All travel-based studies originate from Europe.

© 2020 Willems RPJ et al. *JAMA Intern Med.*

eTable 2. Baseline Characteristics

Study	Mean Age (SD), y[a]	Sex (% female)	Ethnicity or Race (%)[d]	Chronic Disease (%)	Antibiotic Use (%)	Inclusion criteria	Exclusion criteria
Arcilla, 2017	51 (NA)[b]	54%	93% D	23%	7% AT during travel	Age ≥18 years, travelling for ≥1 week and ≤3 months	Age <18 years, incapacitated subjects
Ben-Ami, 2006	76 (NA)[b]	48%	NA	NA	35% AT past 3 months prior to admission	NA	NA
Chanderraj, 2019	58 (NA)[c]	43%	82% W 18% NW	NA	94% AT unspecified	NA	Prior isolation of VRE from sterile site cultures, conversion from VRE negative to positive in <72 hours
Cheng, 2016	59 (24)	44%	NA	51%	NA	According to extensive criteria for opportunistic screening, safety net screening, and extensive contact tracing during hospitalizatio n	NA
Falk, 2000	44 (18)	NA	NA	NA	NA	NA	NA
Ford, 2015	58 (18–86)[b]	41%	NA	NA	NA	NA	NA
Goodman, 2019	55 (15)	46%	46% W 44% B	NA	21% AT past 3 months prior to admission	Age ≥16 years	NA

© 2020 Willems RPJ et al. *JAMA Intern Med.*

Hagel, 2019	67 (16)	56%	NA	NA	NA	Age >18 years, external referral, screened for carriage <48 hours of admission to cardiothoracic surgery, gastroenterology-hepatology-infectology and/or geriatric medicine department, expected LOS >48 hours	Multiple admissions, LOS <48 hours
Hamprecht, 2016	62 (NA)[b]	49%	NA	NA	36% AT past 6 months	Age ≥18 years, admission to general department	Patients from ICUs, dermatology, obstetrics, ophthalmology, otorhinolaryngology and psychiatry
Huizinga, 2017	65 (NA)[b,c]	56%	NA	NA	NA	Age >18 years, LOS <48 hours or received day care	Admission >2 days

© 2020 Willems RPJ et al. *JAMA Intern Med.*

eTable 2. Baseline Characteristics (continued)							
**Study**	**Mean Age (SD), y[a]**	**Sex (% female)**	**Ethnicity or Race (%)[d]**	**Chronic Disease (%)**	**Antibiotic Use (%)**	**Inclusion criteria**	**Exclusion criteria**
Kuenzli, 2014	41 (1–73)[b]	56%	NA	NA	5% AT during travel	Any age, travel to South Asia (India, Bhutan, Nepal, and Sri Lanka)	Travel >5 weeks, travel to other countries than India, Bhutan, Nepal, and Sri Lanka or to more than one of these countries
Latour, 2019	86 (35–109)[b]	76%	NA	69% CCI >2	23% AT past 3 months	NA	NA
Lee, 2018	71 (NA)	75%	NA	NA	27% AT past 3 months	Age ≥ 18 years, clinical diagnosis of UTI at the emergency department	UTI with multiple pathogens
McNeil, 2006	50 (9)	41%	89% W 5% B 3% H 2% A 1% NTA	NA	57% AT past 6 months prior to positive index culture	Age >18 years, on the liver transplantation waiting list at the University of Michigan	NA
Okamoto, 2017	62 (15)	47%	26% W 57% B 11% H 8% O	NA	76% AT after the last negative surveillance culture screening through the estimated date of acquisition	NA	LOS <72 hours, culture positive for KPC-E prior to admission or prior to positive surveillance culture
Östholm-Balkhed, 2013	54 (18-76)[b]	59%	NA	NA	7% AT during travel	Age ≥18 years, travel outside Scandinavia for no more than 3 months	Absence of two complete samples, pre-travel sample submission >4 months before travel, post-travel sample submission >4 months upon return
Prasad, 2016	75 (23–102)[b]	63%	41% W 36% B 10% A 12% H	NA	NA	Adults	Signs and symptoms of

© 2020 Willems RPJ et al. *JAMA Intern Med.*

							diarrhea, Clostridium difficile–associated infection, receiving treatment for Clostridium-difficile infection, residing on the short-term rehabilitation floor
Puzniak, 2001	59 (18)	50%	57% W 43% NW	NA	NA	LOS >24 hours	Unknown VRE status at the time of admission
Reuland, 2016	50 (18–95)[b,c]	61%	90% E	NA	15% AT past 12 months	Age ≥18 years	Terminally ill

© 2020 Willems RPJ et al. *JAMA Intern Med.*

eTable 2. Baseline Characteristics (continued)							
**Study**	**Mean Age (SD), y[a]**	**Sex (% female)**	**Ethnicity or Race (%)[d]**	**Chronic Disease (%)**	**Antibiotic Use (%)**	**Inclusion criteria**	**Exclusion criteria**
Rodríguez-Baño, 2008	40 (NA)[b,c]	60%	NA	30%	10% AT past 2 months	Age >14 years	Previous isolation of ESBL-E
Seekatz, 2018	63 (15)	45%	64% B 36% NB	NA	56% AT between admission and fecal specimen collection	NA	History of acute bacterial or viral colitis (including Clostridium difficile infection), active inflammatory bowel disease <14 days before sample collection, presence of a rectal tube, fecal incontinence device, or colostomy at the time of sample collection
Slaughter, 1996	20% >65 years	33%	80% B 20% O	NA	NA	LOS ≥48 hours	NA
Søgaard, 2017	65 (NA)[b,c]	82%	97% NE	15% CCI >2	70% AT within 31-365 days before index date	Aged 15-85 years	Age <15 years and age >85 years
Tan, 2018	73 (21–106)[b]	46%	77% C	20% CCI >5	NA	Hospital LOS >48 hours, and residents of the care facilities	NA
Vading, 2016	49 (NA)[b]	68%	NA	21%	9% AT during travel	Age ≥18 years, travel to South-East Asia, Indian subcontinent, northern Africa or Middle East	Absence of two complete samples
Wielders, 2017	59 (20–72)[b]	55%	98% D	20%	5% AT past 3 months	Aged 18–70 years, living in the eastern part of Noord-Brabant or the northern part of Limburg, inhabitant of a municipality with <30.000 residents, living <10 km of one of the research centers	Working or living on a farm

© 2020 Willems RPJ et al. *JAMA Intern Med.*

Abbreviations: AT, antibiotic treatment; CCI, Charlson Comorbidity Index; LOS, length of stay; NA, not available; SD, standard deviation.
[a] Data indicate mean (SD) unless otherwise specified.
[b] Data presented as median (range).
[c] Age of corresponding controls, which did not significantly differ from the cases of the individual studies.
[d] Ethnicity: Dutch (D), European (E), Northern-European (NE), Chinese (C); Race: White (W), Black (B), Asian (A), Hispanic (H), Native American (NTA), Other (O), NW (Nonwhite), NB (Nonblack).

© 2020 Willems RPJ et al. *JAMA Intern Med.*

eTable 3. Exposure and Outcome Ascertainment

Study	Selective enrichment	Detection method	Method of antibiotic susceptibility testing	Interpretative guideline of antibiotic susceptibility	Characterization method	Exposure ascertainment
Arcilla, 2017	Y	ESBL-selective chromogenic agar	Automated microdilution system and disk diffusion	NVMM	Genotypic (microarray, PCR and DNA-sequencing)	Structured questionnaires
Ben-Ami, 2006	N	Antibiotic-supplemented MCK agar	Disk diffusion	CLSI	Genotypic (PCR)[b]	Medical records and interviews
Chanderraj, 2019	N	VRE-selective chromogenic agar	Selective agar	NA	Phenotypic	Medical records
Cheng, 2016	Y	Antibiotic-supplemented MCK agar	Disk diffusion	CLSI	Genotypic (PCR)	Information database
Falk, 2000	Y	Bile esculin azide agar	Agar dilution method and Etest	NA	Phenotypic	Medical records
Ford, 2015	N	Bile esculin agar	Automated microdilution system	NA	Phenotypic	Information databases
Goodman, 2019	N	Antibiotic disk-supplemented MCK agar	Disk diffusion and modified carbapenem inactivation method	CLSI	Genotypic (microarray)	Medical records
Hagel, 2019	N	ESBL- and KPC-selective chromogenic agar	Automated microdilution system	NA	Genotypic (microarray)	Medical records
Hamprecht, 2016	N	ESBL-selective chromogenic agar	Automated microdilution system and disk diffusion	EUCAST	Genotypic (PCR)	Structured questionnaires
Huizinga, 2017	Y	ESBL-selective screening agar	Automated microdilution system and disk diffusion	EUCAST, NVMM	Genotypic (microarray)	Medical records and home-medication lists verfied by assistant pharmacist
Kuenzli, 2014	N	ESBL-selective chromogenic agar	Automated microdilution system, Etest, disk diffusion	EUCAST, CLSI	Genotypic (microarray, PCR and DNA-sequencing)[b]	Structured questionnaires

© 2020 Willems RPJ et al. *JAMA Intern Med.*

			and modified Hodge test			
Latour, 2019	N	ESBL-, CPE-, and OXA-48-selective chromogenic agars	Disk diffusion and Carba NP method	CLSI	Genotypic (microarray and PCR)	Structured questionnaires completed by nurse or coordinating physician

© 2020 Willems RPJ et al. *JAMA Intern Med.*

eTable 3. Exposure and Outcome Ascertainment (continued)						
**Study**	**Selective enrichment**	**Detection method**	**Method of antibiotic susceptibility testing**	**Interpretative guideline of antibiotic susceptibility**	**Characterization method**	**Exposure ascertainment**
Lee, 2018	N	Eosin methylene blue agar	Broth microdilution method and disk diffusion	CLSI	Phenotypic	Medical records
McNeil, 2006	Y	Bile esculin azide selective agar	Selective agar	NA	Phenotypic	Medical records and information database
Okamoto, 2017	N	Antibiotic disk-supplemented MCK agar	Automated microdilution system	NA	Genotypic (PCR)	Medical records
Östholm-Balkhed, 2013	N	ESBL-selective chromogenic agar and antibiotic disk-supplemented chromogenic UTI agar	Etest	EUCAST	Genotypic (PCR)	Structured questionnaires
Prasad, 2016	Y	MCK agar	Automated microdilution system and modified Hodge test	CLSI	Genotypic (PCR)	Medical records
Puzniak, 2001	N	Bile esculin azide selective agar	Selective agar	NA	Phenotypic	Medical informatics and information databases
Reuland, 2016	Y	ESBL-selective screening agar	Automated microdilution system and disk diffusion	EUCAST, NVMM	Genotypic (PCR and DNA-sequencing)	Structured questionnaires and information database
Rodríguez-Baño, 2008	N	Antibiotic-supplemented MCK agar	Disk diffusion	CLSI	Genotypic (PCR and DNA-sequencing)	Structured questionnaires
Seekatz, 2018	N	MCK agar	Automated microdilution system	NA	Genotypic (PCR)	Medical records
Slaughter, 1996	N	Bile esculin azide agar and antibiotic-supplemented	Agar dilution method and disk diffusion	NCCLS	Genotypic (PCR)	Medical records

© 2020 Willems RPJ et al. *JAMA Intern Med.*

		Columbia colistin-nalidixic acid agar				
Søgaard, 2017	N	NA	Disk diffusion and Etest	EUCAST, SRGA	Phenotypic	Information database
Tan, 2018	N	VRE-selective chromogenic agar	Automated microdilution system	NR	Genotypic (PCR)	Medical records

© 2020 Willems RPJ et al. *JAMA Intern Med.*

eTable 3. Exposure and Outcome Ascertainment (continued)

Study	Selective enrichment	Detection method	Method of antibiotic susceptibility testing	Interpretative guideline of antibiotic susceptibility	Characterization method	Exposure ascertainment
Vading, 2016	Y[a]	ESBL-selective chromogenic agar	Automated microdilution system and disk diffusion	EUCAST	Genotypic (microarray)	Structured questionnaires
Wielders, 2017	Y	Antibiotic-supplemented MCK agar and E. coli/coliform selective chromogenic agar	Disk diffusion	CLSI	Genotypic (microarray, PCR and DNA-sequencing)	Structured questionnaires and verification of medication use during research centre visits

Abbreviations: CLSI, Clinical and Laboratory Standards Institute; EUCAST, European Committee on Antimicrobial Susceptibility Testing; MCK, MacConkey; NA, not available; N, no; NCCLS, National Committee for Clinical Laboratory Standards; NVMM, Dutch Society for Medical Microbiology; PCR, polymerase chain reaction; SRGA, Swedish Reference Group of Antibiotics; Y, yes.

[a] Selective enrichment was performed for carbapenemase detection only.
[b] Studies in which genotypic characterization was performed for a selected number of isolates that were collected.

© 2020 Willems RPJ et al. *JAMA Intern Med.*

eTable 4. Adjusted Study Results

Study	Outcome[a]	Exposure	OR (95% CI)	Adjustment
Arcilla, 2016	ESBL-E	Acid suppression	1.17 (0.84 – 1.63)[c]	Adjusted for age, sex, antibiotic use, comorbidity, travel
Ben-Ami, 2006	ESBL-E	H2RAs	2.80 (1.10 – 7.40)	Adjusted for antibiotic use, chronic renal insufficiency, liver disease, poor functional status
Cheng, 2016	CPE	PPIs	2.84 (1.72 – 4.71)	Adjusted for age, sex, antibiotic use, prior hospital admission, presence of indwelling device, LTCF residency
Falk, 2000	VRE	Antacids	24.20 (2.90 - ∞)[d]	Adjusted for presence of diarrhea
Hamprecht, 2016	ESBL-E	Acid suppression	1.22 (1.07 – 1.40)	Adjusted for age, sex, antibiotic use, travel, history of MDRO colonization or infection, LTCF stay, hospital center
Huizinga, 2016	ESBL-E	PPIs and H2RAs	3.69 (1.56 – 8.73)[c]	Adjusted for age, sex, antibiotic use, prior hospital admission
		PPIs	3.89 (1.65 – 9.19)[c]	Adjusted for age, sex, antibiotic use, prior hospital admission
Latour, 2019	ESBL-E	PPIs and H2RAs	1.74 (1.22 – 2.48)	Adjusted for antibiotic use, mobility
McNeil, 2006	VRE	PPIs	2.70 (1.20 – 6.10)	Adjusted for antibiotic use, hospital admission in liver unit, ERCP or paracentesis
Reuland, 2016	ESBL-E	PPIs and H2RAs	1.90 (1.10 – 3.30)	Adjusted for age, sex, antibiotic use, travel
		PPIs	1.90 (1.10 – 3.20)	Adjusted for age, sex, antibiotic use, travel
Søgaard, 2017	ESBL-E[b]	PPIs	1.21 (0.91 – 1.60)	Adjusted for age, sex, antibiotic use, comorbidity, cancer, liver diseases, COPD, immunosuppressants, prior inpatient hospital admission, citizenship
Tan, 2018	VRE	PPIs	1.63 (1.24 – 2.15)	Adjusted for age, sex, comorbidity, longer duration of antibiotic therapy, length of stay of >14 days, higher number of beds per room, prior VRE and MRSA carriage, recent surgery, presence of a skin ulcer, use of an indwelling urinary catheter, year of screening, healthcare facility
Wielders, 2017	ESBL-E	PPIs	1.84 (1.05 – 3.23)	Adjusted for age, sex, travel, pace of residence during youth, animal contact during work or study, lived on a farm during childhood, performed jobs on a farm during childhood, dog owner, kept cows or horses for a hobby during recent years, prior visit to a farm, living close to one or more farms.

Abbreviations: CI, confidence interval; CPE, carbapenemase-producing Enterobacterales; ERCP, endoscopic, retrograde cholangiopancreatogram; ESBL-E, extended-spectrum β-lactamase-producing Enterobacterales; H2RA, histamine$_2$ receptor antagonist, LTCF, long-term care facility; MDRO, multidrug-resistant micro-organism; MRSA, methicillin-resistant *Staphylococcus aureus*; PPI, proton pump inhibitor; VRE, vancomycin-resistant enterococci.

[a] Outcome refers to intestinal carriage unless reported otherwise.
[b] Urinary tract infection (surrogate) outcome.
[c] Results of data received from study authors, with more complete confounding adjustments in addition to those presented in the original papers.
[d] Multivariable analysis was conducted using exact logistic regression

© 2020 Willems RPJ et al. *JAMA Intern Med.*

© 2020 Willems RPJ et al. *JAMA Intern Med.*

eTable 5. Quality Assessment According to the Modified Newcastle-Ottawa Scale[a]											
**Study**	**Selection**				**Comparability**		**Exposure/Outcome[b]**			**Overall Score[c]**	**Risk of Bias**
	**S1**	**S2**	**S3**	**S4**	**C1**	**C2**	**E1/O1**	**E2/O2**	**E3/O3**		
**Case-control studies**										**(Max. of 10)**	
Chanderraj et al, 2019	1	1	1	1	0	0	2	1	0	**7**	Low
Cheng et al, 2016	1	0	1	0	1	1	2	1	0	**7**	Low
Falk et al, 2000	1	0	1	1	0	0	2	1	0	**6**	High
Lee et al, 2018	1	1	1	1	1	0	2	1	0	**8**	Low
Okamoto et al, 2017	1	1	1	1	0	0	2	1	0	**7**	Low
Søgaard et al, 2017	1	1	1	1	1	1	2	1	0	**9**	Low
Seekatz et al, 2018	1	1	1	1	0	0	2	1	0	**7**	Low
**Cohort studies**										**(Max. of 10)**	
Arcilla et al, 2016	1	1	1	1	1	0	1	0	1	**7**	Low
Ford et al, 2015	0	1	2	0	0	1	1	0	0	**5**	High
Hagel et al, 2019	1	1	2	1	0	0	1	0	0	**6**	High
Kuenzli et al, 2014	1	1	1	1	0	0	1	0	1	**6**	High
McNeil et al, 2005	0	1	2	0	0	1	1	0	1	**6**	High
Östholm-Balkhed et al, 2013	1	1	1	1	0	0	1	0	1	**6**	High
Puzniak et al, 2001	1	1	2	1	0	0	1	0	1	**7**	Low
Slaughter et al, 1996	1	1	2	1	0	0	1	0	1	**7**	Low
Vading et al, 2016	1	1	1	1	0	0	1	0	1	**6**	High
**Cross-sectional studies**										**(Max. of 7)**	
Ben-Ami et al, 2006	0	1	2	0	0	1	1	0	0	**5**	High
Hamprecht et al, 2016	1	1	1	0	1	1	1	0	0	**6**	Low
Huizinga et al, 2016	1	1	2	0	1	1	1	0	0	**7**	Low
Reuland et al, 2016	1	1	2	0	1	1	1	0	0	**7**	Low
Rodríguez-Baño et al, 2008	1	0	1	0	0	0	1	0	0	**3**	High
Tan et al, 2018	1	1	2	0	1	1	1	0	0	**7**	Low
Wielders et al, 2016	1	1	2	0	1	0	1	0	0	**6**	Low
Prasad et al, 2016	0	1	2	0	0	0	1	0	0	**4**	High
Latour et al, 2019	1	1	1	0	0	1	1	0	0	**5**	High
Goodman et al, 2019	0	1	2	0	1	1	1	0	0	**6**	Low

[a] 0 not applicable; 1 one star allocated; 2 two stars allocated; 0 no star allocated.
[b] Case-control studies are scored on the exposure of interest, whereas cohort and cross-sectional studies are scored on the outcome of interest.

© 2020 Willems RPJ et al. *JAMA Intern Med.*

[c] Cohort and case-control studies with an overall score of ≥7 were judged to be of high quality. Cross-sectional studies with an overall score of ≥6 were judged to be of high quality.	

© 2020 Willems RPJ et al. *JAMA Intern Med.*

eTable 6. Sensitivity Analyses After Exclusion of Studies on Urinary Tract Infections[a]

Meta-analysis[b]	No. of studies	Outcome	Summary OR (95% CI)	I^2
**Primary analysis**				
Sensitivity analysis before exclusion	12	Intestinal carriage + UTI	1.74 (1.40 – 2.16)	68%
Sensitivity analysis after exclusion	11	Intestinal carriage	1.86 (1.46 - 2.37)	70%
**Subgroup, MDRO subtype**				
**MDR-E**				
Sensitivity analysis before exclusion	19	Intestinal carriage + UTI	1.60 (1.33 - 1.92)	54%
Sensitivity analysis after exclusion	17	Intestinal carriage	1.67 (1.35 - 2.07)	58%
**ESBL-E**				
Sensitivity analysis before exclusion	19	Intestinal carriage + UTI	1.43 (1.20 - 1.70)	36%
Sensitivity analysis after exclusion	17	Intestinal carriage	1.51 (1.22 - 1.87)	44%
**CPE**				
Sensitivity analysis before exclusion	5	Intestinal carriage	2.04 (1.34 - 3.10)	53%
Sensitivity analysis after exclusion	N/A	N/A	N/A	N/A
**VRE**				
Sensitivity analysis before exclusion	7	Intestinal carriage	1.97 (1.49 - 2.60)	31%
Sensitivity analysis after exclusion	N/A	N/A	N/A	N/A
**Subgroup, acid suppressant**				
**PPI**				
Sensitivity analysis before exclusion	17	Intestinal carriage + UTI	1.81 (1.52 - 2.16)	33%
Sensitivity analysis after exclusion	15	Intestinal carriage	1.93 (1.66 - 2.25)	7%
**H2RA**				
Sensitivity analysis before exclusion	4	Intestinal carriage	1.33 (0.86 - 2.08)	15%
Sensitivity analysis after exclusion	N/A	N/A	N/A	N/A
**Subgroup, design**				
**Cohort**				
Sensitivity analysis before exclusion	5	Intestinal carriage	2.31 (1.56 - 3.43)	0%
Sensitivity analysis after exclusion	N/A	N/A	N/A	N/A
**Case-control**				
Sensitivity analysis before exclusion	7	Intestinal carriage + UTI	1.64 (1.13 - 2.38)	66%
Sensitivity analysis after exclusion	5	Intestinal carriage	1.93 (1.17 - 3.18)	66%
**Cross-sectional**				
Sensitivity analysis before exclusion	13	Intestinal carriage	1.84 (1.47 - 2.30)	58%
Sensitivity analysis after exclusion	N/A	N/A	N/A	N/A
**Subgroup, setting**				
**Travel**				
Sensitivity analysis before exclusion	4	Intestinal carriage	1.11 (0.82 - 1.50)	0%
Sensitivity analysis after exclusion	N/A	N/A	N/A	N/A
**Community**				
Sensitivity analysis before exclusion	4	Intestinal carriage + UTI	1.41 (1.07 - 1.87)	21%
Sensitivity analysis after exclusion	3	Intestinal carriage	1.69 (1.17 - 2.42)	0%
**At hospital admission**				
Sensitivity analysis before exclusion	4	Intestinal carriage	2.39 (1.17 - 4.87)	82%
Sensitivity analysis after exclusion	N/A	N/A	N/A	N/A
**In-hospital**				
Sensitivity analysis before exclusion	11	Intestinal carriage + UTI	1.98 (1.50 - 2.62)	33%
Sensitivity analysis after exclusion	10	Intestinal carriage	2.04 (1.53 - 2.72)	35%

© 2020 Willems RPJ et al. *JAMA Intern Med.*

eTable 6. Sensitivity Analyses After Exclusion of Studies on Urinary Tract Infections[a] (continued)				
**Meta-analysis**	**No. of studies**	**Outcome**	**Summary OR (95% CI)**	I^2
**Sensitivity analysis**				
**High quality**				
Sensitivity analysis before exclusion	15	Intestinal carriage + UTI	1.64 (1.37 - 1.97)	60%
Sensitivity analysis after exclusion	13	Intestinal carriage	1.74 (1.42 – 2.14)	64%
**High quality with max. NOS score for exposure ascertainment**				
Sensitivity analysis before exclusion	13	Intestinal carriage + UTI	1.81 (1.47 – 2.21)	45%
Sensitivity analysis after exclusion	11	Intestinal carriage	1.94 (1.60 - 2.36)	28%
**High quality with max. NOS score for exposure ascertainment and age, sex, and antibiotic use adjustment**				
Sensitivity analysis before exclusion	5	Intestinal carriage + UTI	1.88 (1.33 - 2.64)	69%
Sensitivity analysis after exclusion	4	Intestinal carriage	2.15 (1.52 – 3.04)	49%

Abbreviations: CI, confidence interval; CPE, carbapenemase-producing *Enterobacterales*; ESBL-E, extended-spectrum β-lactamase-producing *Enterobacterales*; H2RA, histamine$_2$ receptor antagonist; MDR-E, multidrug-resistant *Enterobacterales* (ESBL-E and CPE); MDRO, multidrug-resistant micro-organism; N/A, not applicable; OR, odds ratio; PPI, proton pump inhibitor; UTI, urinary tract infection; VRE, vancomycin-resistant enterococci.
[a] Sensitivity analyses were performed after exclusion of the studies by Søgaard et al. and Lee et al.
[b] Subgroup analyses were performed on all 26 studies.

© 2020 Willems RPJ et al. *JAMA Intern Med.*

eTable 7. Sensitivity Analysis by the Leaving-One-Out Method[a]

Excluded study	Results After Study Exclusion
	**Summary OR (95% CI)**
Arcilla et al, 2016	1.75 (1.48 – 2.07)
Ben-Ami et al, 2006	1.68 (1.43 – 1.97)
Chanderraj et al, 2019	1.70 (1.43 – 2.01)
Cheng et al, 2016	1.64 (1.40 – 1.92)
Falk et al, 2000	1.66 (1.43 – 1.93)
Ford et al, 2015	1.69 (1.44 – 1.99)
Goodman et al, 2019	1.64 (1.40 – 1.92)
Hagel et al, 2019	1.69 (1.43 – 1.99)
Hamprecht et al, 2016	1.75 (1.49 – 2.07)
Huizinga et al, 2016	1.66 (1.41 – 1.94)
Kuenzli et al, 2014	1.71 (1.45 – 2.01)
Latour et al, 2019	1.70 (1.43 – 2.01)
Lee et al, 2018	1.71 (1.45 – 2.01)
McNeil et al, 2005	1.67 (1.42 – 1.96)
Okamoto et al, 2017	1.73 (1.47 – 2.04)
Prasad et al, 2016	1.68 (1.42 – 1.98)
Puzniak et al, 2001	1.67 (1.43 – 1.97)
Reuland et al, 2016	1.69 (1.43 – 1.99)
Rodríguez-Baño et al, 2008	1.72 (1.46 – 2.02)
Seekatz et al, 2018	1.71 (1.45 – 2.01)
Slaughter et al, 1996	1.69 (1.44 – 1.99)
Søgaard et al, 2017	1.75 (1.47 – 2.08)
Tan et al, 2018	1.71 (1.44 – 2.03)
Vading et al, 2016	1.72 (1.47 – 2.02)
Wielders et al, 2016	1.69 (1.43 – 2.00)
Östholm-Balkhed et al, 2013	1.71 (1.45 – 2.01)

Abbreviations: CI, confidence interval; OR, odds ratio.
[a] Overall summary estimate: OR, 1.71; 95% CI, 1.45 – 2.01.

© 2020 Willems RPJ et al. *JAMA Intern Med.*

eTable 8. Sensitivity Analysis by Knapp-Hartung Modification[a]

Included Studies	No. of studies	Coefficient	SE	t	P>|t|	LCL	UCL	I^2	
With studies investigating the proxy outcome	26	0.52	0.09	6.07	<.001	0.34	0.69	53%	
Without studies investigating the proxy outcome[b]	24	0.55	0.09	6.17	<.001	0.37	0.74	55%	

Abbreviations: SE, standard error of the coefficient; LCL, lower confidence limit of the coefficient; UCL, upper confidence limit of the coefficient.
[a] Calculated with STATA software; all values were rounded to the second decimal point.
[b] Sensitivity analysis was performed after exclusion of the studies by Søgaard et al. and Lee et al.

© 2020 Willems RPJ et al. *JAMA Intern Med.*

eFigure 1. Secondary analysis

Abbreviations: AST, acid suppressive therapy; IV, inverse variance (red).

Study or Subgroup	log[Odds Ratio]	SE	Weight	Odds Ratio IV, Random, 95% CI
Arcilla et al, 2016	0.1579	0.1682	7.1%	1.17 [0.84, 1.63]
Ben-Ami et al, 2006	1.0296	0.4863	2.2%	2.80 [1.08, 7.26]
Chanderraj et al, 2019	0.5596	0.1521	7.5%	1.75 [1.30, 2.36]
Cheng et al, 2016	1.0438	0.257	5.1%	2.84 [1.72, 4.70]
Falk et al, 2000	3.1864	1.0825	0.5%	24.20 [2.90, 201.96]
Ford et al, 2015	0.8308	0.6627	1.3%	2.30 [0.63, 8.41]
Goodman et al, 2019	1.2197	0.337	3.7%	3.39 [1.75, 6.55]
Hagel et al, 2019	0.7276	0.363	3.4%	2.07 [1.02, 4.22]
Hamprecht et al, 2016	0.1989	0.0686	9.5%	1.22 [1.07, 1.40]
Huizinga et al, 2016	1.3059	0.4391	2.6%	3.69 [1.56, 8.73]
Kuenzli et al, 2014	−0.1278	0.8755	0.8%	0.88 [0.16, 4.89]
Latour et al, 2019	0.5536	0.181	6.8%	1.74 [1.22, 2.48]
Lee et al, 2018	0	0.6385	1.4%	1.00 [0.29, 3.50]
McNeil et al, 2005	0.9933	0.4148	2.8%	2.70 [1.20, 6.09]
Okamoto et al, 2017	0	0.3626	3.4%	1.00 [0.49, 2.04]
Prasad et al, 2016	0.7419	0.2941	4.4%	2.10 [1.18, 3.74]
Puzniak et al, 2001	1.2751	0.606	1.5%	3.58 [1.09, 11.74]
Reuland et al, 2016	0.6419	0.2803	4.6%	1.90 [1.10, 3.29]
Rodríguez-Baño et al, 2008	−0.0645	0.4764	2.3%	0.94 [0.37, 2.39]
Seekatz et al, 2018	0.3365	0.4224	2.7%	1.40 [0.61, 3.20]
Slaughter et al, 1996	0.6374	0.3995	3.0%	1.89 [0.86, 4.14]
Søgaard et al, 2017	0.1906	0.144	7.7%	1.21 [0.91, 1.60]
Tan et al, 2018	0.4886	0.1404	7.8%	1.63 [1.24, 2.15]
Vading et al, 2016	−0.7733	0.6623	1.3%	0.46 [0.13, 1.69]
Wielders et al, 2016	0.6098	0.2867	4.5%	1.84 [1.05, 3.23]
Östholm-Balkhed et al, 2013	0.2546	0.5284	1.9%	1.29 [0.46, 3.63]
**Total (95% CI)**			**100.0%**	**1.70 [1.44, 1.99]**

Heterogeneity: $Tau^2 = 0.07$; $Chi^2 = 54.13$, df = 25 (P = 0.0006); $I^2 = 54\%$

Test for overall effect: Z = 6.45 (P < 0.00001)

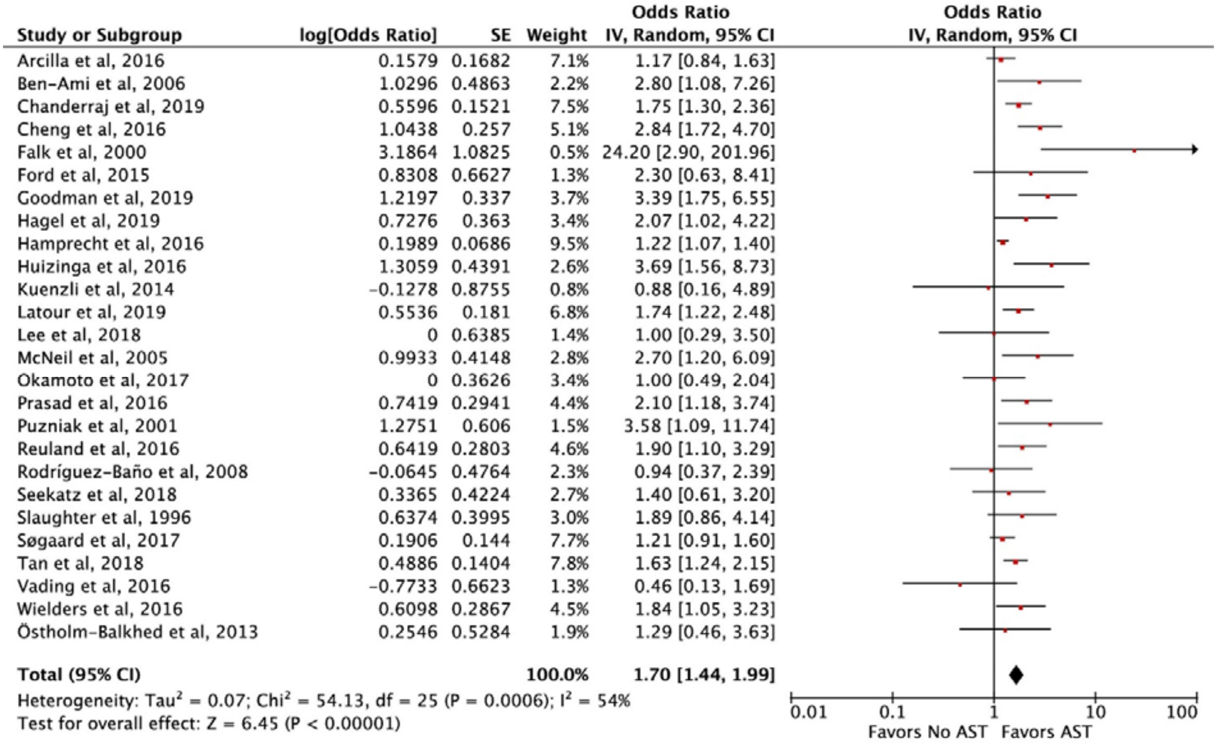

eFigure 2. Subgroup Analysis by Design

Abbreviations: AST, acid suppressive therapy; IV, inverse variance (red).

eFigure 2A. Cohort

Study or Subgroup	log[Odds Ratio]	SE	Weight	Odds Ratio IV, Random, 95% CI
**Adjusted**				
McNeil et al, 2005	0.9933	0.4148	23.6%	2.70 [1.20, 6.09]
**Subtotal (95% CI)**			**23.6%**	**2.70 [1.20, 6.09]**
Heterogeneity: Not applicable				
Test for overall effect: Z = 2.39 (P = 0.02)				
**Unadjusted**				
Ford et al, 2015	0.8308	0.6627	9.2%	2.30 [0.63, 8.41]
Hagel et al, 2019	0.7276	0.363	30.8%	2.07 [1.02, 4.22]
Puzniak et al, 2001	1.2751	0.606	11.0%	3.58 [1.09, 11.74]
Slaughter et al, 1996	0.6374	0.3995	25.4%	1.89 [0.86, 4.14]
**Subtotal (95% CI)**			**76.4%**	**2.20 [1.40, 3.46]**
Heterogeneity: $Tau^2 = 0.00$; $Chi^2 = 0.82$, df = 3 (P = 0.84); $I^2 = 0\%$				
Test for overall effect: Z = 3.43 (P = 0.0006)				
**Total (95% CI)**			**100.0%**	**2.31 [1.56, 3.43]**
Heterogeneity: $Tau^2 = 0.00$; $Chi^2 = 1.01$, df = 4 (P = 0.91); $I^2 = 0\%$				
Test for overall effect: Z = 4.16 (P < 0.0001)				

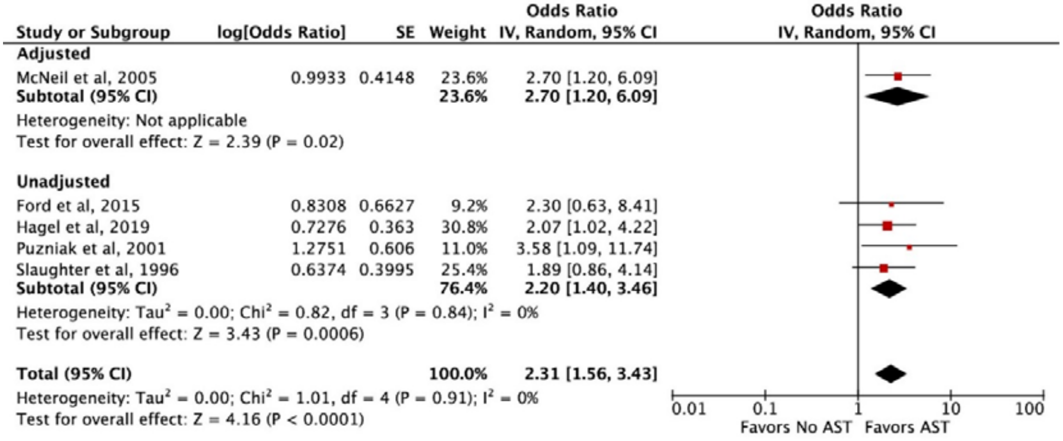

eFigure 2B. Case-control

Study or Subgroup	log[Odds Ratio]	SE	Weight	Odds Ratio IV, Random, 95% CI
**Adjusted**				
Cheng et al, 2016	1.0438	0.257	18.2%	2.84 [1.72, 4.70]
Falk et al, 2000	3.1864	1.0825	2.8%	24.20 [2.90, 201.96]
Søgaard et al, 2017	0.1906	0.144	23.6%	1.21 [0.91, 1.60]
**Subtotal (95% CI)**			**44.6%**	**2.65 [1.00, 6.98]**
Heterogeneity: $Tau^2 = 0.53$; $Chi^2 = 14.95$, df = 2 (P = 0.0006); $I^2 = 87\%$				
Test for overall effect: Z = 1.96 (P = 0.05)				
**Unadjusted**				
Chanderraj et al, 2019	0.5596	0.1521	23.2%	1.75 [1.30, 2.36]
Lee et al, 2018	0	0.6385	6.7%	1.00 [0.29, 3.50]
Okamoto et al, 2017	0	0.3626	13.7%	1.00 [0.49, 2.04]
Seekatz et al, 2018	0.3365	0.4224	11.7%	1.40 [0.61, 3.20]
**Subtotal (95% CI)**			**55.4%**	**1.56 [1.21, 2.01]**
Heterogeneity: $Tau^2 = 0.00$; $Chi^2 = 2.63$, df = 3 (P = 0.45); $I^2 = 0\%$				
Test for overall effect: Z = 3.40 (P = 0.0007)				
**Total (95% CI)**			**100.0%**	**1.64 [1.13, 2.38]**
Heterogeneity: $Tau^2 = 0.13$; $Chi^2 = 17.58$, df = 6 (P = 0.007); $I^2 = 66\%$				
Test for overall effect: Z = 2.58 (P = 0.010)				

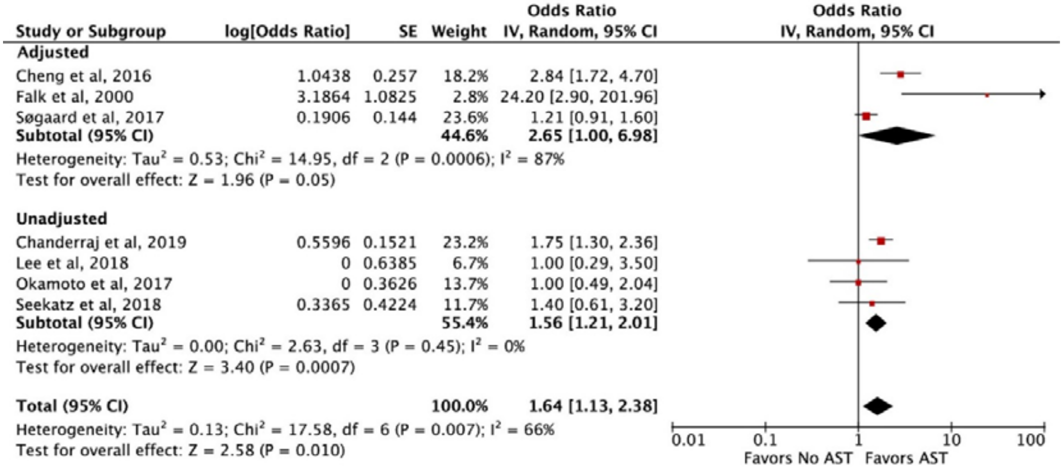

eFigure 2C. Cross-sectional

Study or Subgroup	log[Odds Ratio]	SE	Weight	Odds Ratio IV, Random, 95% CI
**Adjusted**				
Ben-Ami et al, 2006	1.0296	0.4863	4.2%	2.80 [1.08, 7.26]
Hamprecht et al, 2016	0.1989	0.0686	17.0%	1.22 [1.07, 1.40]
Huizinga et al, 2016	1.3059	0.4391	4.9%	3.69 [1.56, 8.73]
Latour et al, 2019	0.5536	0.181	12.4%	1.74 [1.22, 2.48]
Reuland et al, 2016	0.6419	0.2803	8.6%	1.90 [1.10, 3.29]
Tan et al, 2018	0.4886	0.1404	14.2%	1.63 [1.24, 2.15]
Wielders et al, 2016	0.6098	0.2867	8.4%	1.84 [1.05, 3.23]
**Subtotal (95% CI)**			**69.7%**	**1.71 [1.34, 2.18]**
Heterogeneity: $Tau^2 = 0.05$; $Chi^2 = 15.64$, df = 6 (P = 0.02); $I^2 = 62\%$				
Test for overall effect: Z = 4.30 (P < 0.0001)				
**Unadjusted**				
Goodman et al, 2019	1.2197	0.337	7.0%	3.39 [1.75, 6.55]
Hagel et al, 2019	0.7276	0.363	6.3%	2.07 [1.02, 4.22]
Kuenzli et al, 2014	−0.1278	0.8755	1.5%	0.88 [0.16, 4.89]
Prasad et al, 2016	0.7419	0.2941	8.2%	2.10 [1.18, 3.74]
Puzniak et al, 2001	1.2751	0.606	2.9%	3.58 [1.09, 11.74]
Rodríguez-Baño et al, 2008	−0.0645	0.4764	4.3%	0.94 [0.37, 2.39]
**Subtotal (95% CI)**			**30.3%**	**2.11 [1.43, 3.11]**
Heterogeneity: $Tau^2 = 0.06$; $Chi^2 = 6.62$, df = 5 (P = 0.25); $I^2 = 24\%$				
Test for overall effect: Z = 3.74 (P = 0.0002)				
**Total (95% CI)**			**100.0%**	**1.84 [1.47, 2.30]**
Heterogeneity: $Tau^2 = 0.07$; $Chi^2 = 28.26$, df = 12 (P = 0.005); $I^2 = 58\%$				
Test for overall effect: Z = 5.38 (P < 0.00001)				

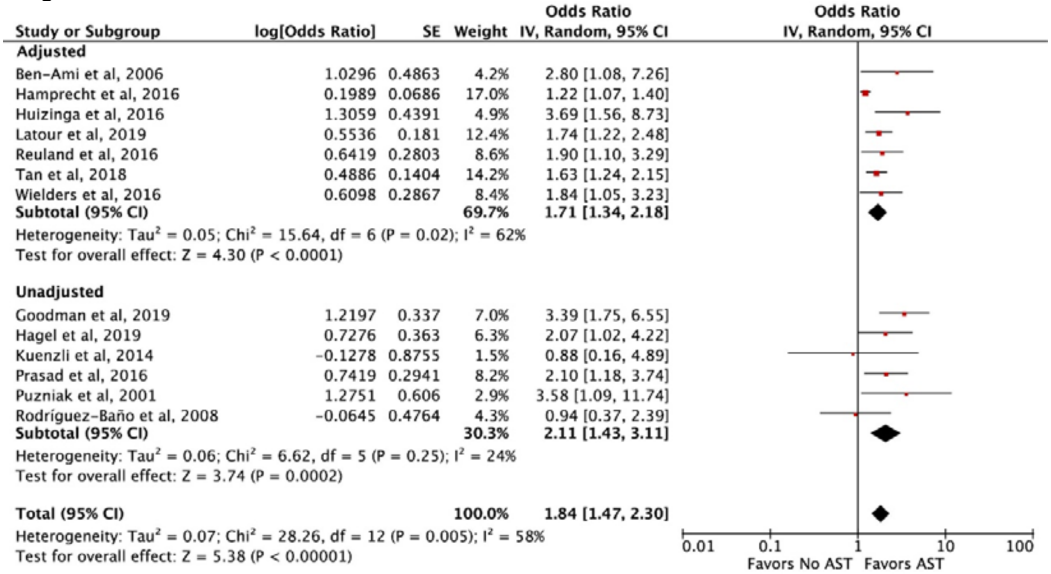

© 2020 Willems RPJ et al. *JAMA Intern Med.*

eFigure 3. Subgroup Analysis by Type of Acid Suppressant

Abbreviations: AST, acid suppressive therapy; IV, inverse variance (red).

eFigure 3A. Proton pump inhibitor

Study or Subgroup	log[Odds Ratio]	SE	Weight	Odds Ratio IV, Random, 95% CI
**Adjusted**				
Cheng et al, 2016	1.0438	0.257	7.6%	2.84 [1.72, 4.70]
Huizinga et al, 2016	1.3584	0.438	3.4%	3.89 [1.65, 9.18]
Latour et al, 2019	0.5536	0.181	11.2%	1.74 [1.22, 2.48]
McNeil et al, 2005	0.9933	0.4148	3.8%	2.70 [1.20, 6.09]
Reuland et al, 2016	0.6419	0.2724	7.0%	1.90 [1.11, 3.24]
Søgaard et al, 2017	0.1906	0.144	13.4%	1.21 [0.91, 1.60]
Tan et al, 2018	0.5539	0.181	11.2%	1.74 [1.22, 2.48]
Wielders et al, 2016	0.6098	0.2867	6.6%	1.84 [1.05, 3.23]
**Subtotal (95% CI)**			**64.1%**	**1.89 [1.49, 2.41]**
Heterogeneity: Tau^2 = 0.06; Chi^2 = 14.62, df = 7 (P = 0.04); I^2 = 52%				
Test for overall effect: Z = 5.17 (P < 0.00001)				
**Unadjusted**				
Ben-Ami et al, 2006	−0.3567	0.5743	2.1%	0.70 [0.23, 2.16]
Chanderraj et al, 2019	0.5596	0.1521	12.9%	1.75 [1.30, 2.36]
Ford et al, 2015	0.8308	0.6627	1.7%	2.30 [0.63, 8.41]
Goodman et al, 2019	1.2197	0.337	5.2%	3.39 [1.75, 6.55]
Kuenzli et al, 2014	−0.1278	0.8755	1.0%	0.88 [0.16, 4.89]
Lee et al, 2018	0	0.6385	1.8%	1.00 [0.29, 3.50]
Prasad et al, 2016	0.7419	0.2941	6.3%	2.10 [1.18, 3.74]
Seekatz et al, 2018	0.3365	0.4224	3.6%	1.40 [0.61, 3.20]
Vading et al, 2016 [7]	−0.1204	0.7624	1.3%	0.89 [0.20, 3.95]
**Subtotal (95% CI)**			**35.9%**	**1.75 [1.33, 2.30]**
Heterogeneity: Tau^2 = 0.03; Chi^2 = 9.38, df = 8 (P = 0.31); I^2 = 15%				
Test for overall effect: Z = 4.02 (P < 0.0001)				
**Total (95% CI)**			**100.0%**	**1.81 [1.52, 2.16]**
Heterogeneity: Tau^2 = 0.04; Chi^2 = 24.04, df = 16 (P = 0.09); I^2 = 33%				
Test for overall effect: Z = 6.71 (P < 0.00001)				

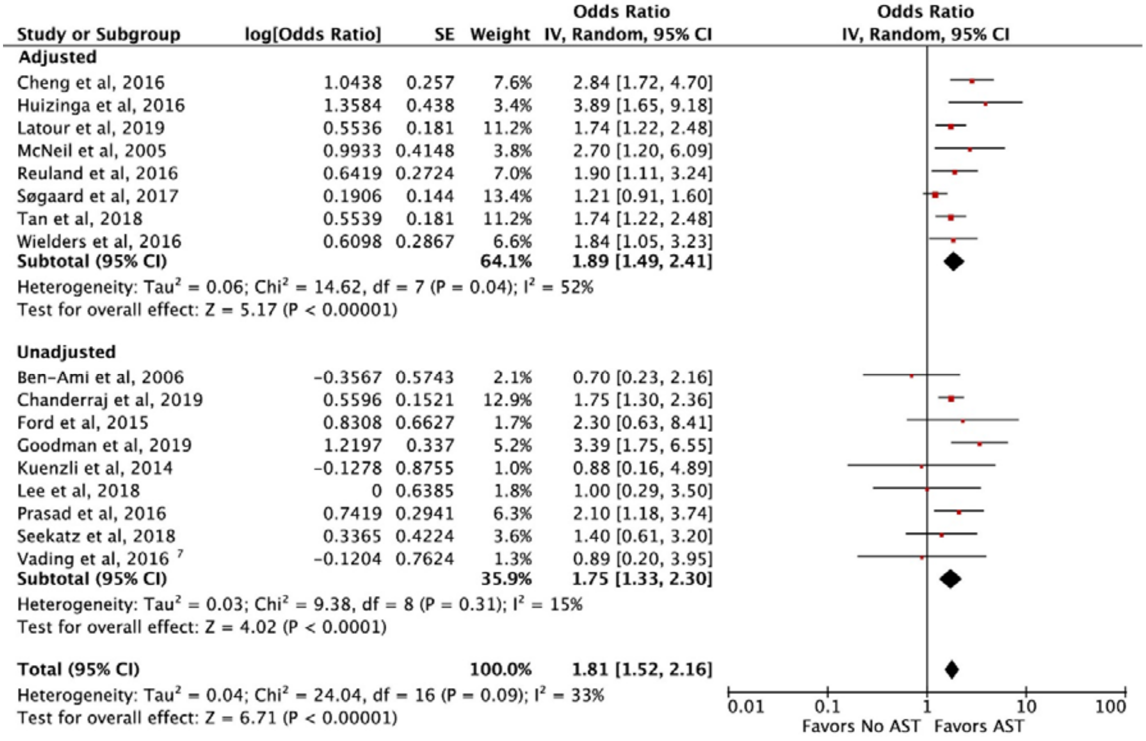

eFigure 3B. Histamine$_2$ receptor antagonist

Study or Subgroup	log[Odds Ratio]	SE	Weight	Odds Ratio IV, Random, 95% CI
**Adjusted**				
Ben-Ami et al, 2006	1.0296	0.4863	17.7%	2.80 [1.08, 7.26]
**Subtotal (95% CI)**			**17.7%**	**2.80 [1.08, 7.26]**
Heterogeneity: Not applicable				
Test for overall effect: Z = 2.12 (P = 0.03)				
**Unadjusted**				
Huizinga et al, 2016	−0.1206	1.4627	2.3%	0.89 [0.05, 15.58]
Reuland et al, 2016	0.5878	1.1031	4.0%	1.80 [0.21, 15.64]
Tan et al, 2018	0.1106	0.1258	75.9%	1.12 [0.87, 1.43]
**Subtotal (95% CI)**			**82.3%**	**1.12 [0.88, 1.43]**
Heterogeneity: Tau^2 = 0.00; Chi^2 = 0.21, df = 2 (P = 0.90); I^2 = 0%				
Test for overall effect: Z = 0.92 (P = 0.36)				
**Total (95% CI)**			**100.0%**	**1.33 [0.86, 2.08]**
Heterogeneity: Tau^2 = 0.05; Chi^2 = 3.53, df = 3 (P = 0.32); I^2 = 15%				
Test for overall effect: Z = 1.27 (P = 0.20)				

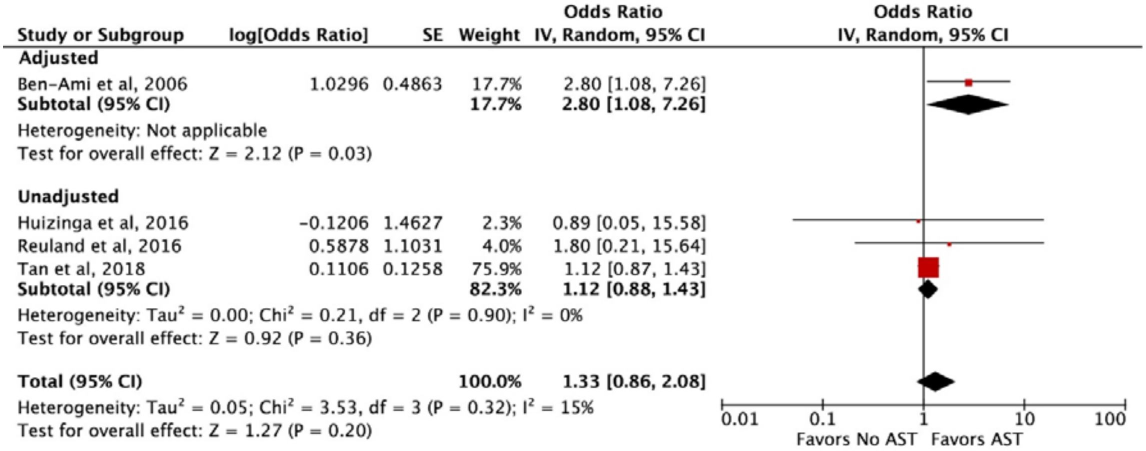

© 2020 Willems RPJ et al. *JAMA Intern Med.*

eFigure 4. Subgroup Analysis by Setting

Abbreviations: AST, acid suppressive therapy; IV, inverse variance (red).

eFigure 4A. Travel

Study or Subgroup	log[Odds Ratio]	SE	Weight	Odds Ratio IV, Random, 95% CI
**Adjusted**				
Arcilla et al, 2016	0.1579	0.1682	83.1%	1.17 [0.84, 1.63]
**Subtotal (95% CI)**			**83.1%**	**1.17 [0.84, 1.63]**
Heterogeneity: Not applicable				
Test for overall effect: Z = 0.94 (P = 0.35)				
**Unadjusted**				
Kuenzli et al, 2014	−0.1278	0.8755	3.1%	0.88 [0.16, 4.89]
Vading et al, 2016	−0.7733	0.6623	5.4%	0.46 [0.13, 1.69]
Östholm-Balkhed et al, 2013	0.2546	0.5284	8.4%	1.29 [0.46, 3.63]
**Subtotal (95% CI)**			**16.9%**	**0.87 [0.42, 1.80]**
Heterogeneity: $Tau^2 = 0.00$; $Chi^2 = 1.47$, df = 2 (P = 0.48); $I^2 = 0\%$				
Test for overall effect: Z = 0.38 (P = 0.70)				
**Total (95% CI)**			**100.0%**	**1.11 [0.82, 1.50]**
Heterogeneity: $Tau^2 = 0.00$; $Chi^2 = 2.01$, df = 3 (P = 0.57); $I^2 = 0\%$				
Test for overall effect: Z = 0.70 (P = 0.48)				

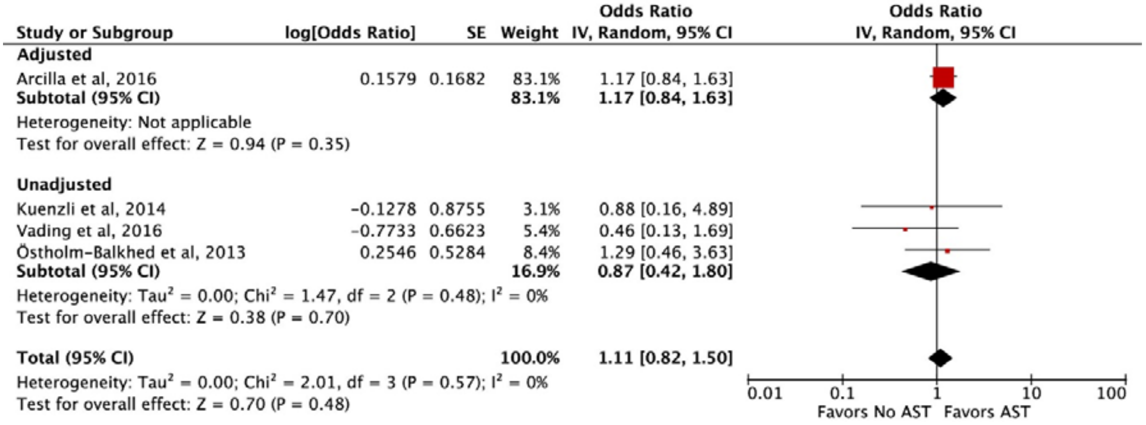

eFigure 4B. Community

Study or Subgroup	log[Odds Ratio]	SE	Weight	Odds Ratio IV, Random, 95% CI
**Adjusted**				
Reuland et al, 2016	0.6419	0.2803	20.7%	1.90 [1.10, 3.29]
Søgaard et al, 2017	0.1906	0.144	51.2%	1.21 [0.91, 1.60]
Wielders et al, 2016	0.6098	0.2867	19.9%	1.84 [1.05, 3.23]
**Subtotal (95% CI)**			**91.8%**	**1.49 [1.08, 2.05]**
Heterogeneity: $Tau^2 = 0.03$; $Chi^2 = 3.12$, df = 2 (P = 0.21); $I^2 = 36\%$				
Test for overall effect: Z = 2.45 (P = 0.01)				
**Unadjusted**				
Rodríguez-Baño et al, 2008	−0.0645	0.4764	8.2%	0.94 [0.37, 2.39]
**Subtotal (95% CI)**			**8.2%**	**0.94 [0.37, 2.39]**
Heterogeneity: Not applicable				
Test for overall effect: Z = 0.14 (P = 0.89)				
**Total (95% CI)**			**100.0%**	**1.41 [1.07, 1.87]**
Heterogeneity: $Tau^2 = 0.02$; $Chi^2 = 3.80$, df = 3 (P = 0.28); $I^2 = 21\%$				
Test for overall effect: Z = 2.45 (P = 0.01)				

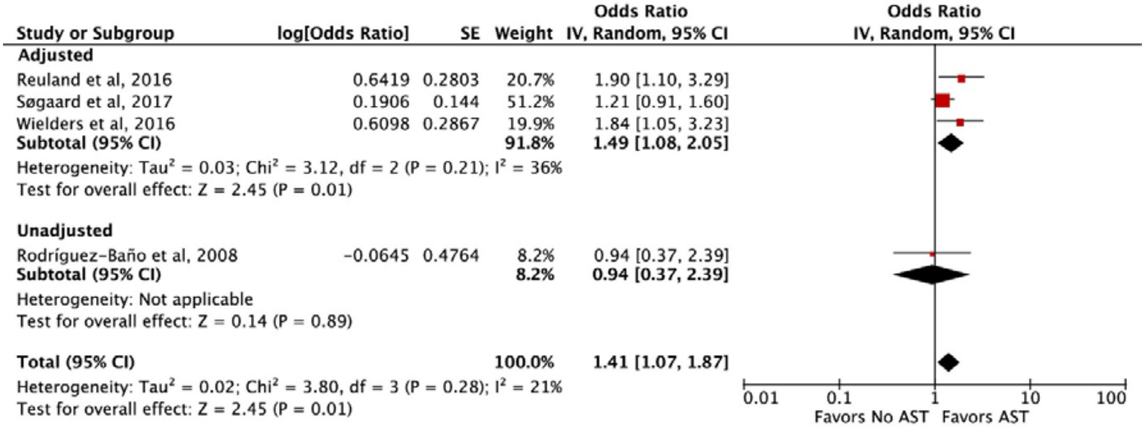

eFigure 4C. At hospital admission

Study or Subgroup	log[Odds Ratio]	SE	Weight	Odds Ratio IV, Random, 95% CI
**Adjusted**				
Ben-Ami et al, 2006	1.0296	0.4863	20.5%	2.80 [1.08, 7.26]
Hamprecht et al, 2016	0.1989	0.0686	32.1%	1.22 [1.07, 1.40]
Huizinga et al, 2016	1.3059	0.4391	22.0%	3.69 [1.56, 8.73]
**Subtotal (95% CI)**			**74.6%**	**2.11 [0.95, 4.71]**
Heterogeneity: $Tau^2 = 0.38$; $Chi^2 = 8.89$, df = 2 (P = 0.01); $I^2 = 77\%$				
Test for overall effect: Z = 1.83 (P = 0.07)				
**Unadjusted**				
Goodman et al, 2019	1.2197	0.337	25.4%	3.39 [1.75, 6.55]
**Subtotal (95% CI)**			**25.4%**	**3.39 [1.75, 6.55]**
Heterogeneity: Not applicable				
Test for overall effect: Z = 3.62 (P = 0.0003)				
**Total (95% CI)**			**100.0%**	**2.39 [1.17, 4.87]**
Heterogeneity: $Tau^2 = 0.41$; $Chi^2 = 17.01$, df = 3 (P = 0.0007); $I^2 = 82\%$				
Test for overall effect: Z = 2.40 (P = 0.02)				

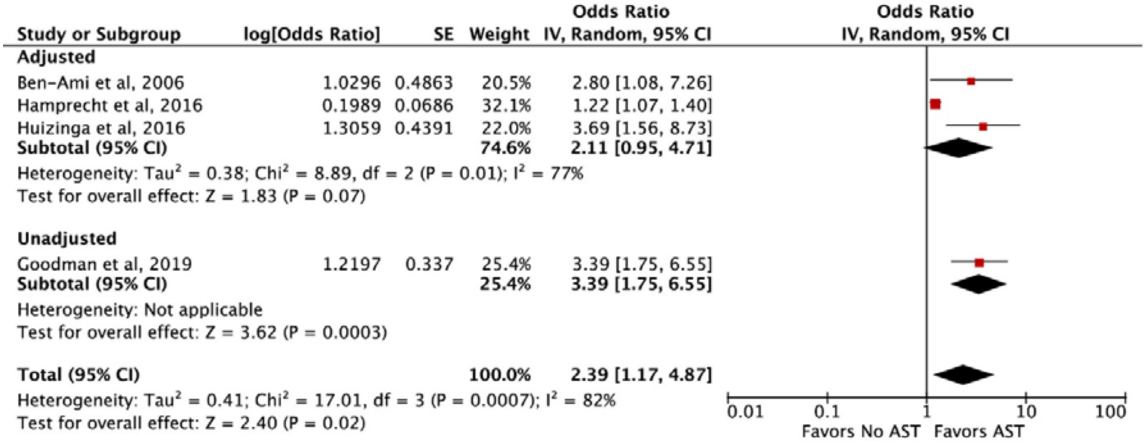

© 2020 Willems RPJ et al. *JAMA Intern Med.*

eFigure 4D. In-hospital

Study or Subgroup	log[Odds Ratio]	SE	Weight	Odds Ratio IV, Random, 95% CI
**Adjusted**				
Cheng et al, 2016	1.0438	0.257	15.5%	2.84 [1.72, 4.70]
Falk et al, 2000	3.1864	1.0825	1.6%	24.20 [2.90, 201.96]
McNeil et al, 2005	0.9933	0.4148	8.6%	2.70 [1.20, 6.09]
**Subtotal (95% CI)**			**25.7%**	**3.44 [1.68, 7.06]**
Heterogeneity: $Tau^2 = 0.19$; $Chi^2 = 3.82$, df = 2 (P = 0.15); $I^2 = 48\%$				
Test for overall effect: Z = 3.38 (P = 0.0007)				
**Unadjusted**				
Chanderraj et al, 2019	0.5596	0.1521	23.2%	1.75 [1.30, 2.36]
Ford et al, 2015	0.8308	0.6627	4.0%	2.30 [0.63, 8.41]
Hagel et al, 2019	0.7276	0.363	10.3%	2.07 [1.02, 4.22]
Lee et al, 2018	0	0.6385	4.3%	1.00 [0.29, 3.50]
Okamoto et al, 2017	0	0.3626	10.3%	1.00 [0.49, 2.04]
Puzniak et al, 2001	1.2751	0.606	4.7%	3.58 [1.09, 11.74]
Seekatz et al, 2018	0.3365	0.4224	8.3%	1.40 [0.61, 3.20]
Slaughter et al, 1996	0.6374	0.3995	9.0%	1.89 [0.86, 4.14]
**Subtotal (95% CI)**			**74.3%**	**1.69 [1.36, 2.12]**
Heterogeneity: $Tau^2 = 0.00$; $Chi^2 = 5.16$, df = 7 (P = 0.64); $I^2 = 0\%$				
Test for overall effect: Z = 4.64 (P < 0.00001)				
**Total (95% CI)**			**100.0%**	**1.98 [1.50, 2.62]**
Heterogeneity: $Tau^2 = 0.06$; $Chi^2 = 14.86$, df = 10 (P = 0.14); $I^2 = 33\%$				
Test for overall effect: Z = 4.81 (P < 0.00001)				

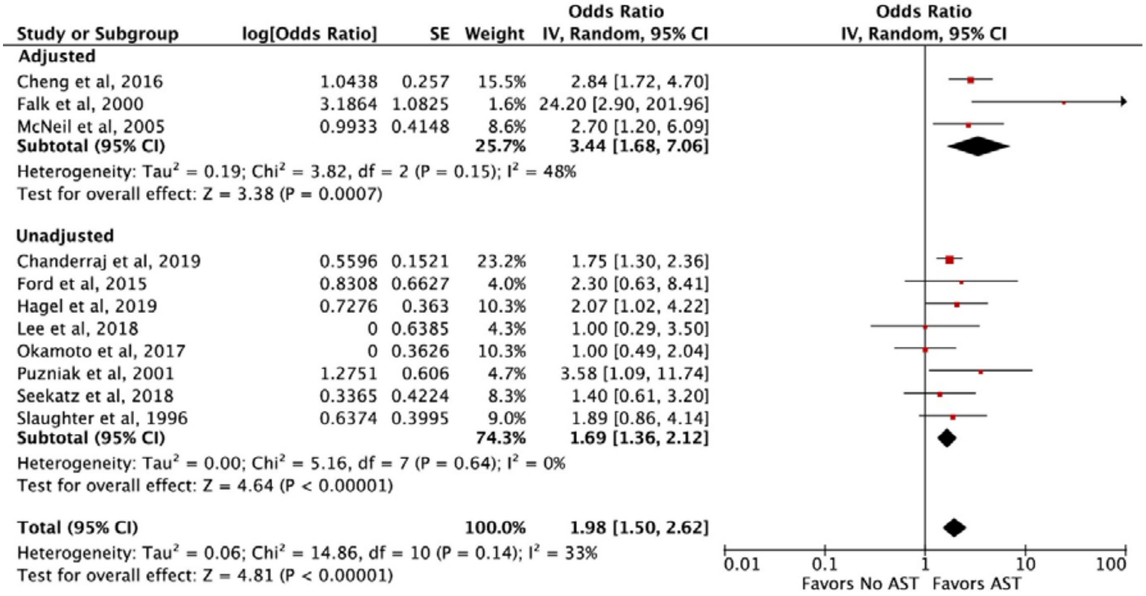

© 2020 Willems RPJ et al. *JAMA Intern Med.*

eFigure 5. Sensitivity Analysis of Studies With High Quality

Abbreviations: AST, acid suppressive therapy; IV, inverse variance (red).

Study or Subgroup	log[Odds Ratio]	SE	Weight	Odds Ratio IV, Random, 95% CI
**Adjusted**				
Arcilla et al, 2016	0.1579	0.1682	9.9%	1.17 [0.84, 1.63]
Cheng et al, 2016	1.0438	0.257	6.9%	2.84 [1.72, 4.70]
Hamprecht et al, 2016	0.1989	0.0686	13.6%	1.22 [1.07, 1.40]
Huizinga et al, 2016	1.3059	0.4391	3.5%	3.69 [1.56, 8.73]
Reuland et al, 2016	0.6419	0.2803	6.3%	1.90 [1.10, 3.29]
Søgaard et al, 2017	0.1906	0.144	10.9%	1.21 [0.91, 1.60]
Tan et al, 2018	0.4886	0.1404	11.0%	1.63 [1.24, 2.15]
Wielders et al, 2016	0.6098	0.2867	6.2%	1.84 [1.05, 3.23]
**Subtotal (95% CI)**			**68.3%**	**1.58 [1.27, 1.96]**
Heterogeneity: $Tau^2 = 0.06$; $Chi^2 = 21.53$, df = 7 (P = 0.003); $I^2 = 67\%$				
Test for overall effect: Z = 4.11 (P < 0.0001)				
**Unadjusted**				
Chanderraj et al, 2019	0.5596	0.1521	10.6%	1.75 [1.30, 2.36]
Goodman et al, 2019	1.2197	0.337	5.0%	3.39 [1.75, 6.55]
Lee et al, 2018	0	0.6385	1.9%	1.00 [0.29, 3.50]
Okamoto et al, 2017	0	0.3626	4.6%	1.00 [0.49, 2.04]
Puzniak et al, 2001	1.2751	0.606	2.0%	3.58 [1.09, 11.74]
Seekatz et al, 2018	0.3365	0.4224	3.7%	1.40 [0.61, 3.20]
Slaughter et al, 1996	0.6374	0.3995	4.0%	1.89 [0.86, 4.14]
**Subtotal (95% CI)**			**31.7%**	**1.79 [1.30, 2.46]**
Heterogeneity: $Tau^2 = 0.05$; $Chi^2 = 8.67$, df = 6 (P = 0.19); $I^2 = 31\%$				
Test for overall effect: Z = 3.57 (P = 0.0004)				
**Total (95% CI)**			**100.0%**	**1.64 [1.37, 1.97]**
Heterogeneity: $Tau^2 = 0.06$; $Chi^2 = 34.93$, df = 14 (P = 0.002); $I^2 = 60\%$				
Test for overall effect: Z = 5.33 (P < 0.00001)				

Odds Ratio
IV, Random, 95% CI

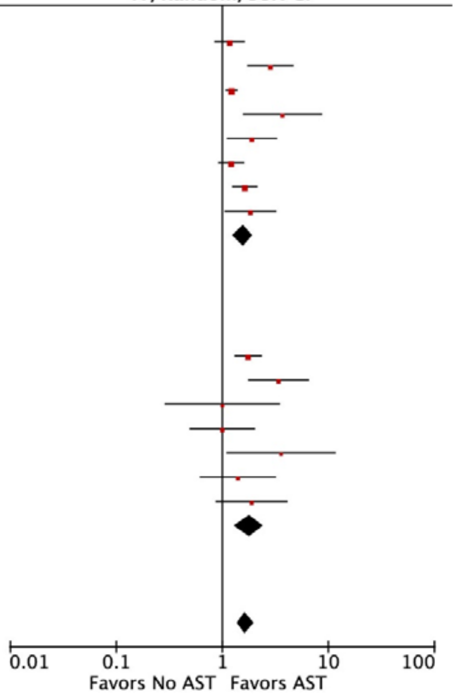

© 2020 Willems RPJ et al. *JAMA Intern Med.*

eFigure 6. Sensitivity Analysis by Mantel-Haenszel Weighting

eFigure 6A. Subgroup analysis by MDRO subtype

Abbreviations: AST, acid suppressive therapy; CPE, carbapenemase-producing Enterobacterales; ESBL-E, extended-spectrum β-lactamase-producing Enterobacterales; MDR-E, multidrug-resistant Enterobacterales; M-H, Mantel-Haenszel (blue); VRE, vancomycin-resistant enterococci.
Subgroup analysis by type of MDRO using the Mantel-Haenszel method with the random effects model.

MDR-E

Study or Subgroup	Acid Suppression Events	Acid Suppression Total	No Acid Suppression Events	No Acid Suppression Total	Weight	Odds Ratio M-H, Random, 95% CI
Arcilla et al, 2016	84	232	527	1558	9.1%	1.11 [0.83, 1.48]
Ben-Ami et al, 2006	15	104	11	137	3.7%	1.93 [0.85, 4.40]
Cheng et al, 2016	60	165	40	335	7.0%	4.21 [2.67, 6.66]
Goodman et al, 2019	17	611	19	2267	4.9%	3.39 [1.75, 6.56]
Hagel et al, 2019	31	306	11	213	4.5%	2.07 [1.02, 4.22]
Hamprecht et al, 2016	186	1640	230	2736	10.1%	1.39 [1.14, 1.71]
Huizinga et al, 2016	22	266	9	304	3.9%	2.96 [1.34, 6.54]
Kuenzli et al, 2014	4	6	114	164	1.1%	0.88 [0.16, 4.95]
Latour et al, 2019	92	622	63	750	8.4%	1.89 [1.35, 2.66]
Lee et al, 2018	4	12	46	138	2.0%	1.00 [0.29, 3.49]
Okamoto et al, 2017	81	162	19	38	4.5%	1.00 [0.49, 2.03]
Prasad et al, 2016	31	119	26	182	5.6%	2.11 [1.18, 3.79]
Reuland et al, 2016	29	202	100	1320	7.1%	2.05 [1.31, 3.18]
Rodríguez-Baño et al, 2008	9	39	16	66	3.1%	0.94 [0.37, 2.38]
Seekatz et al, 2018	13	45	19	86	3.7%	1.43 [0.63, 3.26]
Søgaard et al, 2017	115	950	224	2779	9.7%	1.57 [1.24, 1.99]
Vading et al, 2016	3	16	53	159	1.9%	0.46 [0.13, 1.69]
Wielders et al, 2016	23	288	86	2144	6.7%	2.08 [1.29, 3.35]
Östholm-Balkhed et al, 2013	6	17	62	209	2.7%	1.29 [0.46, 3.65]
**Total (95% CI)**		**5802**		**15585**	**100.0%**	**1.73 [1.42, 2.10]**
Total events	825		1675			

Heterogeneity: Tau^2 = 0.09; Chi^2 = 44.90, df = 18 (P = 0.0004); I^2 = 60%
Test for overall effect: Z = 5.51 (P < 0.00001)

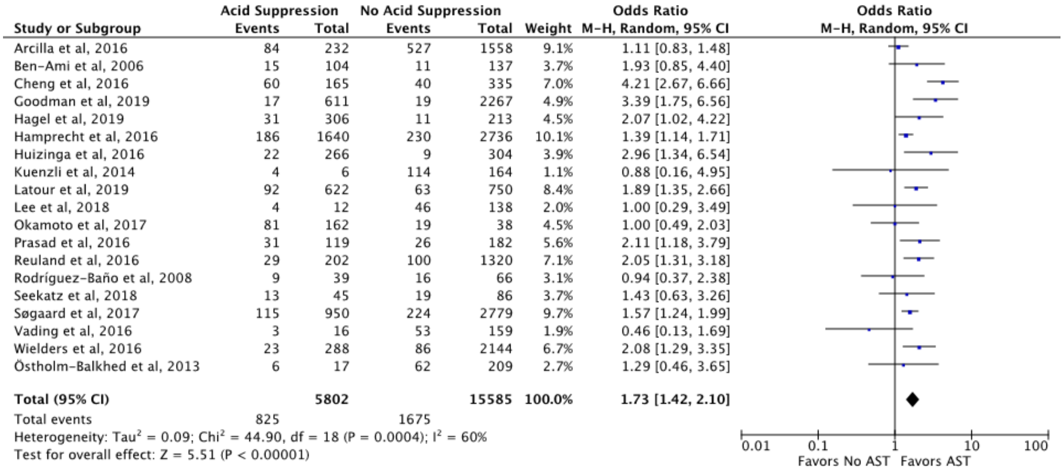

VRE

Study or Subgroup	Acid Suppression Events	Acid Suppression Total	No Acid Suppression Events	No Acid Suppression Total	Weight	Odds Ratio M-H, Random, 95% CI
Chanderraj et al, 2019	445	839	106	263	22.6%	1.67 [1.26, 2.22]
Falk et al, 2000	11	14	4	27	6.0%	21.08 [4.01, 110.93]
Ford et al, 2015	70	73	122	134	8.5%	2.30 [0.63, 8.41]
McNeil et al, 2005	38	174	12	201	16.1%	4.40 [2.22, 8.73]
Puzniak et al, 2001	39	602	3	158	9.5%	3.58 [1.09, 11.74]
Slaughter et al, 1996	34	101	11	52	14.6%	1.89 [0.86, 4.14]
Tan et al, 2018	466	3834	57	1523	22.6%	3.56 [2.68, 4.72]
**Total (95% CI)**		**5637**		**2358**	**100.0%**	**3.04 [1.90, 4.85]**
Total events	1103		315			

Heterogeneity: Tau^2 = 0.23; Chi^2 = 23.82, df = 6 (P = 0.0006); I^2 = 75%
Test for overall effect: Z = 4.65 (P < 0.00001)

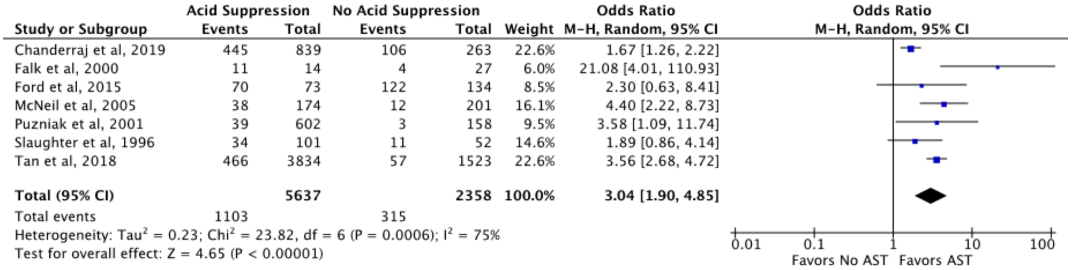

CPE

Study or Subgroup	Acid Suppression Events	Acid Suppression Total	No Acid Suppression Events	No Acid Suppression Total	Weight	Odds Ratio M-H, Random, 95% CI
Cheng et al, 2016	60	165	40	335	23.4%	4.21 [2.67, 6.66]
Goodman et al, 2019	17	611	19	2267	19.7%	3.39 [1.75, 6.56]
Okamoto et al, 2017	81	162	19	38	18.9%	1.00 [0.49, 2.03]
Prasad et al, 2016	31	119	26	182	21.1%	2.11 [1.18, 3.79]
Seekatz et al, 2018	13	45	19	86	16.9%	1.43 [0.63, 3.26]
**Total (95% CI)**		**1102**		**2908**	**100.0%**	**2.22 [1.30, 3.78]**
Total events	202		123			

Heterogeneity: Tau^2 = 0.26; Chi^2 = 14.37, df = 4 (P = 0.006); I^2 = 72%
Test for overall effect: Z = 2.92 (P = 0.003)

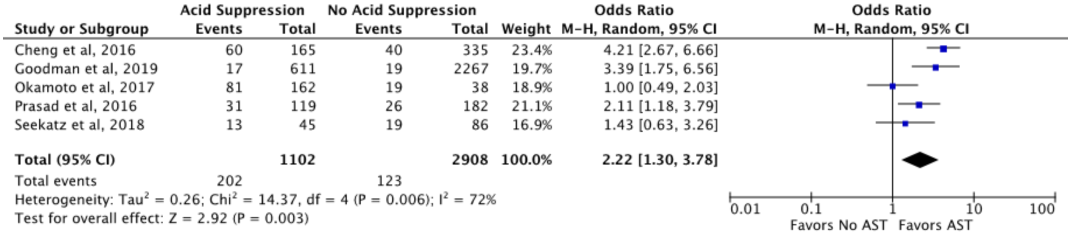

ESBL-E

Study or Subgroup	Acid Suppression Events	Acid Suppression Total	No Acid Suppression Events	No Acid Suppression Total	Weight	Odds Ratio M-H, Random, 95% CI
Arcilla et al, 2016	84	232	527	1558	14.6%	1.11 [0.83, 1.48]
Ben-Ami et al, 2006	15	104	11	137	3.3%	1.93 [0.85, 4.40]
Hagel et al, 2019	31	306	11	213	4.3%	2.07 [1.02, 4.22]
Hamprecht et al, 2016	186	1640	230	2736	19.0%	1.39 [1.14, 1.71]
Huizinga et al, 2016	22	266	9	304	3.6%	2.96 [1.34, 6.54]
Kuenzli et al, 2014	4	6	114	164	0.8%	0.88 [0.16, 4.95]
Latour et al, 2019	92	622	63	750	12.3%	1.89 [1.35, 2.66]
Lee et al, 2018	4	12	46	138	1.6%	1.00 [0.29, 3.49]
Reuland et al, 2016	29	202	100	1320	8.9%	2.05 [1.31, 3.18]
Rodríguez-Baño et al, 2008	9	39	16	66	2.7%	0.94 [0.37, 2.38]
Søgaard et al, 2017	115	950	224	2779	17.1%	1.57 [1.24, 1.99]
Vading et al, 2016	3	16	53	159	1.5%	0.46 [0.13, 1.69]
Wielders et al, 2016	23	288	86	2144	8.0%	2.08 [1.29, 3.35]
Östholm-Balkhed et al, 2013	6	17	62	209	2.2%	1.29 [0.46, 3.65]
**Total (95% CI)**		**4700**		**12677**	**100.0%**	**1.55 [1.32, 1.82]**
Total events	623		1552			

Heterogeneity: Tau^2 = 0.02; Chi^2 = 19.24, df = 13 (P = 0.12); I^2 = 32%
Test for overall effect: Z = 5.37 (P < 0.00001)

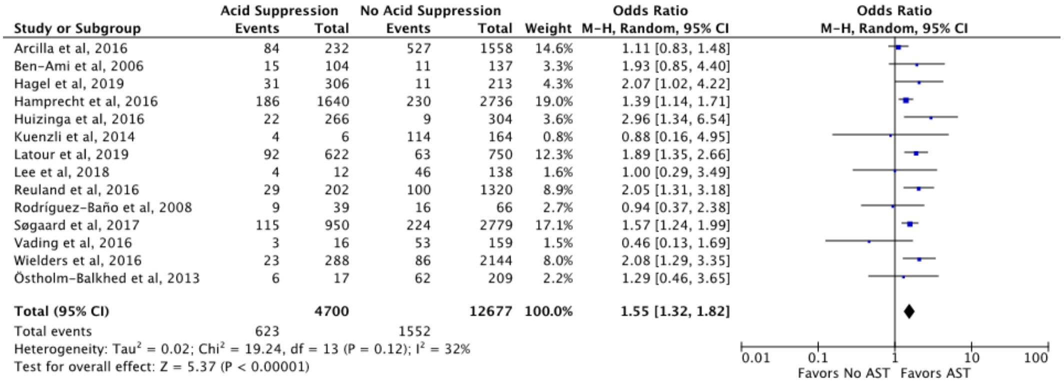

© 2020 Willems RPJ et al. *JAMA Intern Med.*

eFigure 6B. Subgroup analysis by design

Abbreviations: AST, acid suppressive therapy; M-H, Mantel-Haenszel (blue).

Subgroup analysis by study design using the Mantel-Haenszel method with the random effects model.

Cohort

Study or Subgroup	Acid Suppression Events	Acid Suppression Total	No Acid Suppression Events	No Acid Suppression Total	Weight	Odds Ratio M-H, Random, 95% CI
Arcilla et al, 2016	84	232	527	1558	19.5%	1.11 [0.83, 1.48]
Ford et al, 2015	70	73	122	134	7.7%	2.30 [0.63, 8.41]
Hagel et al, 2019	31	306	11	213	13.9%	2.07 [1.02, 4.22]
Kuenzli et al, 2014	4	6	114	164	5.2%	0.88 [0.16, 4.95]
McNeil et al, 2005	38	174	12	201	14.3%	4.40 [2.22, 8.73]
Puzniak et al, 2001	39	602	3	158	8.6%	3.58 [1.09, 11.74]
Slaughter et al, 1996	34	101	11	52	13.0%	1.89 [0.86, 4.14]
Vading et al, 2016	3	16	53	159	7.7%	0.46 [0.13, 1.69]
Östholm-Balkhed et al, 2013	6	17	62	209	10.0%	1.29 [0.46, 3.65]
**Total (95% CI)**		**1527**		**2848**	**100.0%**	**1.73 [1.10, 2.72]**
Total events	309		915			

Heterogeneity: $Tau^2 = 0.25$; $Chi^2 = 20.94$, df = 8 (P = 0.007); $I^2 = 62\%$

Test for overall effect: Z = 2.38 (P = 0.02)

Odds Ratio M-H, Random, 95% CI

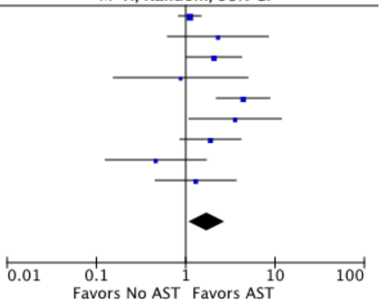

Case-control

Study or Subgroup	Acid Suppression Events	Acid Suppression Total	No Acid Suppression Events	No Acid Suppression Total	Weight	Odds Ratio M-H, Random, 95% CI
Chanderraj et al, 2019	445	839	106	263	21.0%	1.67 [1.26, 2.22]
Cheng et al, 2016	60	165	40	335	18.2%	4.21 [2.67, 6.66]
Falk et al, 2000	11	14	4	27	5.1%	21.08 [4.01, 110.93]
Lee et al, 2018	4	12	46	138	7.7%	1.00 [0.29, 3.49]
Okamoto et al, 2017	81	162	19	38	14.1%	1.00 [0.49, 2.03]
Seekatz et al, 2018	13	45	19	86	12.4%	1.43 [0.63, 3.26]
Søgaard et al, 2017	115	950	224	2779	21.5%	1.57 [1.24, 1.99]
**Total (95% CI)**		**2187**		**3666**	**100.0%**	**1.95 [1.27, 2.98]**
Total events	729		458			

Heterogeneity: $Tau^2 = 0.20$; $Chi^2 = 27.01$, df = 6 (P = 0.0001); $I^2 = 78\%$

Test for overall effect: Z = 3.08 (P = 0.002)

Odds Ratio M-H, Random, 95% CI

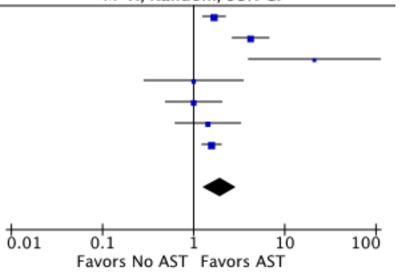

Cross-sectional

Study or Subgroup	Acid Suppression Events	Acid Suppression Total	No Acid Suppression Events	No Acid Suppression Total	Weight	Odds Ratio M-H, Random, 95% CI
Ben-Ami et al, 2006	15	104	11	137	6.7%	1.93 [0.85, 4.40]
Goodman et al, 2019	17	611	19	2267	8.4%	3.39 [1.75, 6.56]
Hamprecht et al, 2016	186	1640	230	2736	14.5%	1.39 [1.14, 1.71]
Huizinga et al, 2016	22	266	9	304	7.0%	2.96 [1.34, 6.54]
Latour et al, 2019	92	622	63	750	12.7%	1.89 [1.35, 2.66]
Prasad et al, 2016	31	119	26	182	9.4%	2.11 [1.18, 3.79]
Reuland et al, 2016	29	202	100	1320	11.3%	2.05 [1.31, 3.18]
Rodríguez-Baño et al, 2008	9	39	16	66	5.8%	0.94 [0.37, 2.38]
Tan et al, 2018	466	3834	57	1523	13.5%	3.56 [2.68, 4.72]
Wielders et al, 2016	23	288	86	2144	10.8%	2.08 [1.29, 3.35]
**Total (95% CI)**		**7725**		**11429**	**100.0%**	**2.12 [1.60, 2.80]**
Total events	890		617			

Heterogeneity: $Tau^2 = 0.13$; $Chi^2 = 34.66$, df = 9 (P < 0.0001); $I^2 = 74\%$

Test for overall effect: Z = 5.21 (P < 0.00001)

Odds Ratio M-H, Random, 95% CI

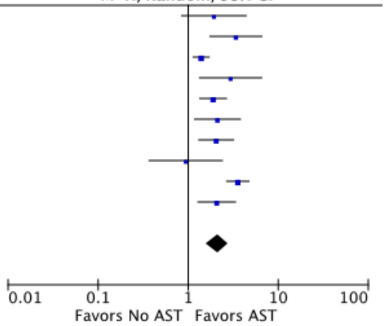

© 2020 Willems RPJ et al. *JAMA Intern Med.*

eFigure 6C. Subgroup analysis by acid suppressant

Abbreviations: AST, acid suppressive therapy; M-H, Mantel-Haenszel (blue).Subgroup analysis by type of acid suppressant using the Mantel-Haenszel method with the random effects model.

Proton pump inhibitor

Study or Subgroup	Acid Suppression Events	Acid Suppression Total	No Acid Suppression Events	No Acid Suppression Total	Weight	Odds Ratio M-H, Random, 95% CI
Ben-Ami et al, 2006	5	59	21	182	4.2%	0.71 [0.26, 1.97]
Chanderraj et al, 2019	445	839	106	263	9.7%	1.67 [1.26, 2.22]
Cheng et al, 2016	60	165	40	335	8.3%	4.21 [2.67, 6.66]
Ford et al, 2015	70	73	122	134	3.0%	2.30 [0.63, 8.41]
Goodman et al, 2019	17	611	19	2267	6.5%	3.39 [1.75, 6.56]
Huizinga et al, 2016	22	259	9	311	5.5%	3.11 [1.41, 6.89]
Kuenzli et al, 2014	4	6	114	164	1.9%	0.88 [0.16, 4.95]
Lee et al, 2018	4	12	46	138	3.2%	1.00 [0.29, 3.49]
McNeil et al, 2005	38	174	12	201	6.3%	4.40 [2.22, 8.73]
Prasad et al, 2016	31	119	26	182	7.2%	2.11 [1.18, 3.79]
Reuland et al, 2016	27	188	102	1334	8.3%	2.03 [1.29, 3.19]
Seekatz et al, 2018	13	45	19	86	5.4%	1.43 [0.63, 3.26]
Søgaard et al, 2017	115	950	224	2779	10.0%	1.57 [1.24, 1.99]
Tan et al, 2018	448	3461	75	1896	9.9%	3.61 [2.81, 4.64]
Vading et al, 2016	2	15	54	160	2.4%	0.30 [0.07, 1.39]
Wielders et al, 2016	23	288	86	2144	8.1%	2.08 [1.29, 3.35]
**Total (95% CI)**		**7264**		**12576**	**100.0%**	**2.11 [1.62, 2.76]**
Total events	1324		1075			

Heterogeneity: $Tau^2 = 0.17$; $Chi^2 = 55.63$, df = 15 (P < 0.00001); $I^2 = 73\%$

Test for overall effect: Z = 5.50 (P < 0.00001)

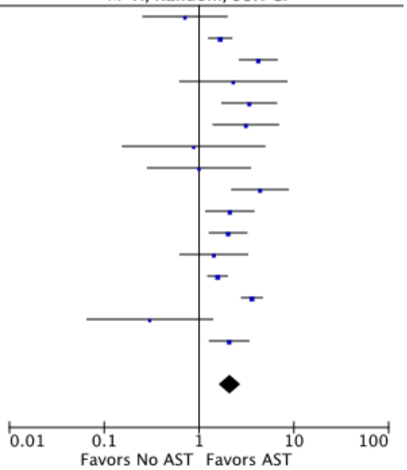

Histamine₂ receptor antagonist

Study or Subgroup	Acid Suppression Events	Acid Suppression Total	No Acid Suppression Events	No Acid Suppression Total	Weight	Odds Ratio M-H, Random, 95% CI
Ben-Ami et al, 2006	10	45	16	196	30.5%	3.21 [1.35, 7.67]
Huizinga et al, 2016	0	9	31	561	5.2%	0.89 [0.05, 15.58]
Reuland et al, 2016	1	7	128	1515	8.9%	1.81 [0.22, 15.12]
Tan et al, 2018	84	791	439	4566	55.3%	1.12 [0.87, 1.43]
**Total (95% CI)**		**852**		**6838**	**100.0%**	**1.59 [0.80, 3.16]**
Total events	95		614			

Heterogeneity: $Tau^2 = 0.21$; $Chi^2 = 5.45$, df = 3 (P = 0.14); $I^2 = 45\%$

Test for overall effect: Z = 1.32 (P = 0.19)

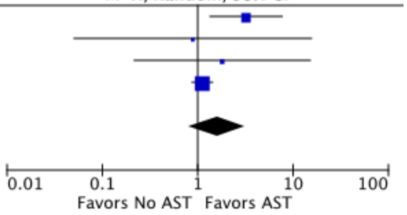

© 2020 Willems RPJ et al. *JAMA Intern Med.*

eFigure 6D. Subgroup analysis by setting

Abbreviations: AST, acid suppressive therapy; M-H, Mantel-Haenszel (blue).
Subgroup analysis by study setting using the Mantel-Haenszel method with the random effects model.

Travel

Study or Subgroup	Acid Suppression Events	Acid Suppression Total	No Acid Suppression Events	No Acid Suppression Total	Weight	Odds Ratio M-H, Random, 95% CI
Arcilla et al, 2016	84	232	527	1558	86.7%	1.11 [0.83, 1.48]
Kuenzli et al, 2014	4	6	114	164	2.4%	0.88 [0.16, 4.95]
Vading et al, 2016	3	16	53	159	4.3%	0.46 [0.13, 1.69]
Östholm-Balkhed et al, 2013	6	17	62	209	6.7%	1.29 [0.46, 3.65]
**Total (95% CI)**		**271**		**2090**	**100.0%**	**1.07 [0.82, 1.40]**
Total events	97		756			

Heterogeneity: $Tau^2 = 0.00$; $Chi^2 = 1.86$, df = 3 (P = 0.60); $I^2 = 0\%$
Test for overall effect: Z = 0.53 (P = 0.60)

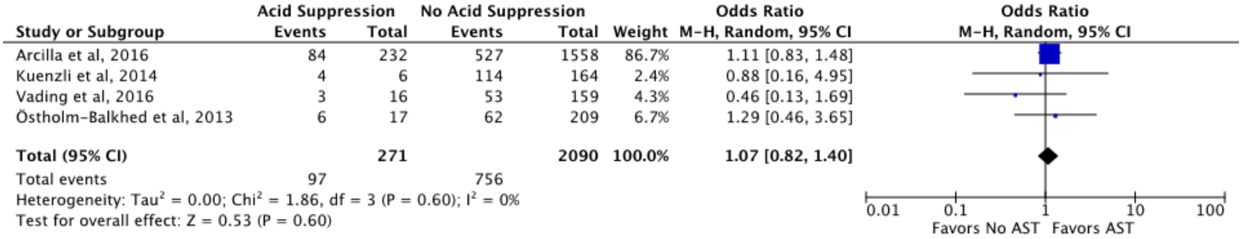

Community

Study or Subgroup	Acid Suppression Events	Acid Suppression Total	No Acid Suppression Events	No Acid Suppression Total	Weight	Odds Ratio M-H, Random, 95% CI
Reuland et al, 2016	29	202	100	1320	20.4%	2.05 [1.31, 3.18]
Rodríguez-Baño et al, 2008	9	39	16	66	5.0%	0.94 [0.37, 2.38]
Søgaard et al, 2017	115	950	224	2779	56.9%	1.57 [1.24, 1.99]
Wielders et al, 2016	23	288	86	2144	17.8%	2.08 [1.29, 3.35]
**Total (95% CI)**		**1479**		**6309**	**100.0%**	**1.70 [1.38, 2.10]**
Total events	176		426			

Heterogeneity: $Tau^2 = 0.01$; $Chi^2 = 3.33$, df = 3 (P = 0.34); $I^2 = 10\%$
Test for overall effect: Z = 4.93 (P < 0.00001)

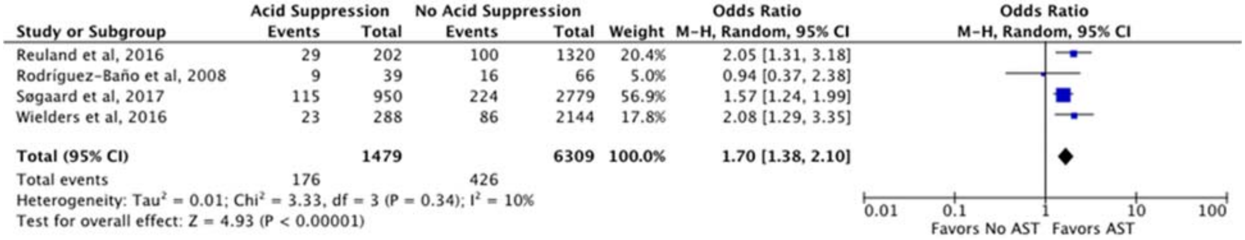

At hospital admission

Study or Subgroup	Acid Suppression Events	Acid Suppression Total	No Acid Suppression Events	No Acid Suppression Total	Weight	Odds Ratio M-H, Random, 95% CI
Ben-Ami et al, 2006	15	104	11	137	19.3%	1.93 [0.85, 4.40]
Goodman et al, 2019	17	611	19	2267	23.6%	3.39 [1.75, 6.56]
Hamprecht et al, 2016	186	1640	230	2736	36.9%	1.39 [1.14, 1.71]
Huizinga et al, 2016	22	266	9	304	20.1%	2.96 [1.34, 6.54]
**Total (95% CI)**		**2621**		**5444**	**100.0%**	**2.13 [1.28, 3.54]**
Total events	240		269			

Heterogeneity: $Tau^2 = 0.17$; $Chi^2 = 9.19$, df = 3 (P = 0.03); $I^2 = 67\%$
Test for overall effect: Z = 2.91 (P = 0.004)

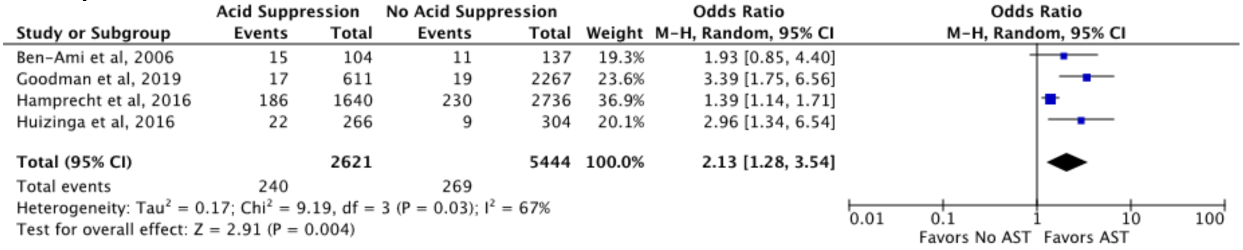

In-hospital

Study or Subgroup	Acid Suppression Events	Acid Suppression Total	No Acid Suppression Events	No Acid Suppression Total	Weight	Odds Ratio M-H, Random, 95% CI
Chanderraj et al, 2019	445	839	106	263	14.7%	1.67 [1.26, 2.22]
Cheng et al, 2016	60	165	40	335	13.0%	4.21 [2.67, 6.66]
Falk et al, 2000	11	14	4	27	4.0%	21.08 [4.01, 110.93]
Ford et al, 2015	70	73	122	134	5.7%	2.30 [0.63, 8.41]
Hagel et al, 2019	31	306	11	213	10.3%	2.07 [1.02, 4.22]
Lee et al, 2018	4	12	46	138	6.0%	1.00 [0.29, 3.49]
McNeil et al, 2005	38	174	12	201	10.6%	4.40 [2.22, 8.73]
Okamoto et al, 2017	81	162	19	38	10.4%	1.00 [0.49, 2.03]
Puzniak et al, 2001	39	602	3	158	6.4%	3.58 [1.09, 11.74]
Seekatz et al, 2018	13	45	19	86	9.3%	1.43 [0.63, 3.26]
Slaughter et al, 1996	34	101	11	52	9.6%	1.89 [0.86, 4.14]
**Total (95% CI)**		**2493**		**1645**	**100.0%**	**2.32 [1.58, 3.41]**
Total events	826		393			

Heterogeneity: $Tau^2 = 0.24$; $Chi^2 = 30.54$, df = 10 (P = 0.0007); $I^2 = 67\%$
Test for overall effect: Z = 4.27 (P < 0.0001)

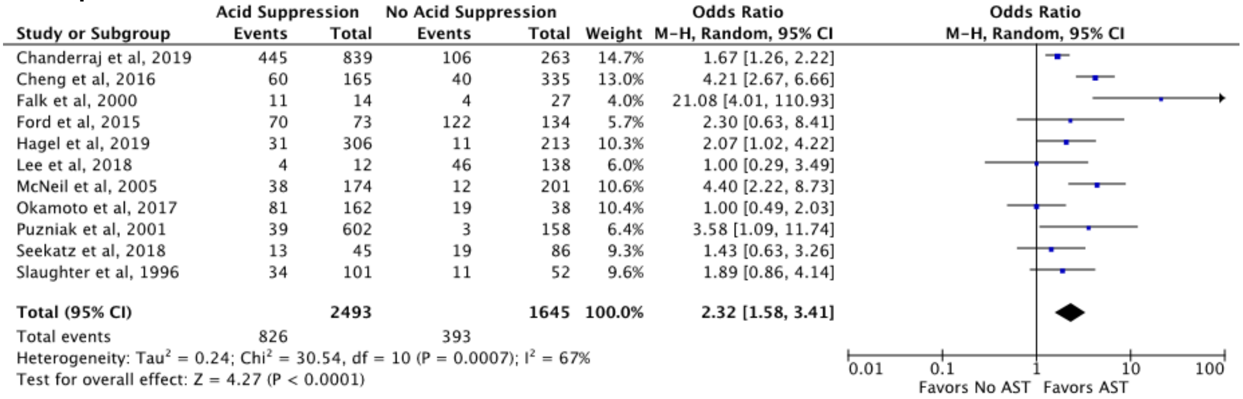

© 2020 Willems RPJ et al. *JAMA Intern Med.*

eFigure 6E. Secondary Analysis

Abbreviations: AST, acid suppressive therapy; M-H, Mantel-Haenszel (blue).
Secondary analysis of all studies using the Mantel-Haenszel method with the random effects model.

Study or Subgroup	Acid Suppression Events	Acid Suppression Total	No Acid Suppression Events	No Acid Suppression Total	Weight	Odds Ratio M–H, Random, 95% CI
Arcilla et al, 2016[8]	84	232	527	1558	6.1%	1.11 [0.83, 1.48]
Ben-Ami et al, 2006[37]	15	104	11	137	3.2%	1.93 [0.85, 4.40]
Chanderraj et al, 2019[38]	445	839	106	263	6.2%	1.67 [1.26, 2.22]
Cheng et al, 2016[39]	60	165	40	335	5.1%	4.21 [2.67, 6.66]
Falk et al, 2000[40]	11	14	4	27	1.2%	21.08 [4.01, 110.93]
Ford et al, 2015[41]	70	73	122	134	1.8%	2.30 [0.63, 8.41]
Goodman et al, 2019[42]	17	611	19	2267	4.0%	3.39 [1.75, 6.56]
Hagel et al, 2019[43]	31	306	11	213	3.7%	2.07 [1.02, 4.22]
Hamprecht et al, 2016[44]	186	1640	230	2736	6.5%	1.39 [1.14, 1.71]
Huizinga et al, 2016[9]	22	266	9	304	3.3%	2.96 [1.34, 6.54]
Kuenzli et al, 2014[45]	4	6	114	164	1.1%	0.88 [0.16, 4.95]
Latour et al, 2019[46]	92	622	63	750	5.8%	1.89 [1.35, 2.66]
Lee et al, 2018[47]	4	12	46	138	1.9%	1.00 [0.29, 3.49]
McNeil et al, 2005[48]	38	174	12	201	3.8%	4.40 [2.22, 8.73]
Okamoto et al, 2017[15]	81	162	19	38	3.7%	1.00 [0.49, 2.03]
Prasad et al, 2016[50]	31	119	26	182	4.4%	2.11 [1.18, 3.79]
Puzniak et al, 2001[51]	39	602	3	158	2.0%	3.58 [1.09, 11.74]
Reuland et al, 2016[10]	29	202	100	1320	5.2%	2.05 [1.31, 3.18]
Rodríguez-Baño et al, 2008[52]	9	39	16	66	2.8%	0.94 [0.37, 2.38]
Seekatz et al, 2018[53]	13	45	19	86	3.2%	1.43 [0.63, 3.26]
Slaughter et al, 1996[54]	34	101	11	52	3.4%	1.89 [0.86, 4.14]
Søgaard et al, 2017[55]	115	950	224	2779	6.4%	1.57 [1.24, 1.99]
Tan et al, 2018[56]	466	3834	57	1523	6.2%	3.56 [2.68, 4.72]
Vading et al, 2016[57]	3	16	53	159	1.8%	0.46 [0.13, 1.69]
Wielders et al, 2016[58]	23	288	86	2144	5.0%	2.08 [1.29, 3.35]
Östholm-Balkhed et al, 2013[49]	6	17	62	209	2.4%	1.29 [0.46, 3.65]
**Total (95% CI)**		**11439**		**17943**	**100.0%**	**1.96 [1.60, 2.39]**
Total events	1928		1990			

Heterogeneity: $Tau^2 = 0.15$; $Chi^2 = 88.10$, df = 25 ($P < 0.00001$); $I^2 = 72\%$
Test for overall effect: Z = 6.63 ($P < 0.00001$)

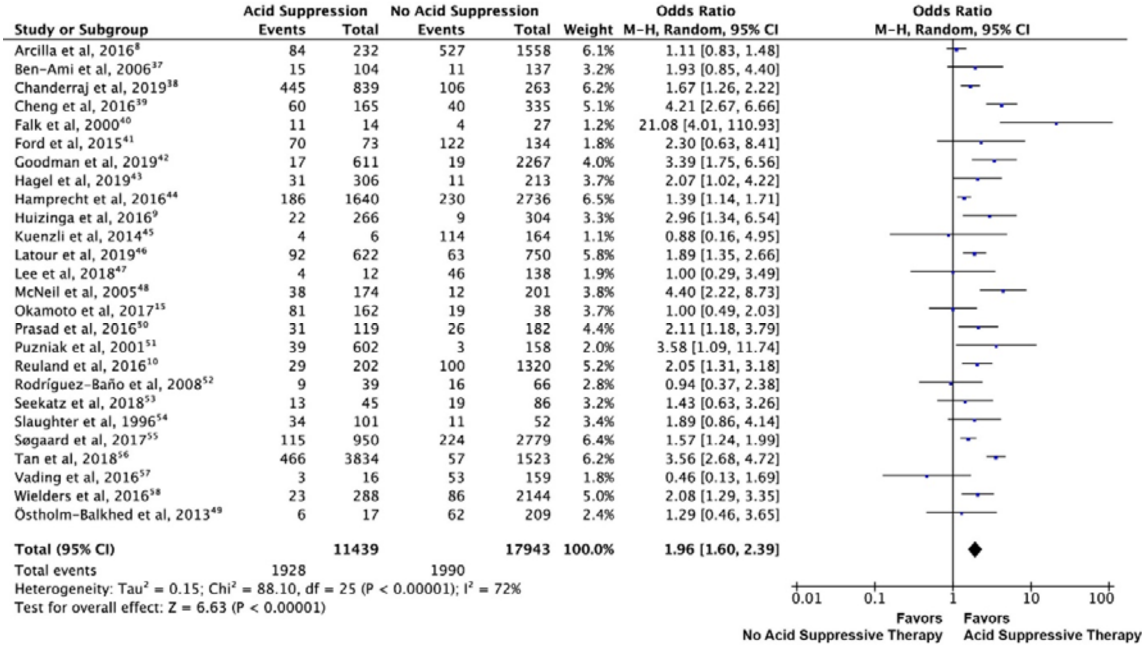

eFigure 6F. Analysis of studies with high quality

Abbreviations: AST, acid suppressive therapy; M-H, Mantel-Haenszel (blue).
Analysis of the high-quality studies using the Mantel-Haenszel method with the random effects model.

Study or Subgroup	Acid Suppression Events	Acid Suppression Total	No Acid Suppression Events	No Acid Suppression Total	Weight	Odds Ratio M–H, Random, 95% CI
Arcilla et al, 2016	84	232	527	1558	9.0%	1.11 [0.83, 1.48]
Chanderraj et al, 2019	445	839	106	263	9.0%	1.67 [1.26, 2.22]
Cheng et al, 2016	60	165	40	335	7.5%	4.21 [2.67, 6.66]
Goodman et al, 2019	17	611	19	2267	5.8%	3.39 [1.75, 6.56]
Hamprecht et al, 2016	186	1640	230	2736	9.6%	1.39 [1.14, 1.71]
Huizinga et al, 2016	22	266	9	304	4.9%	2.96 [1.34, 6.54]
Lee et al, 2018	4	12	46	138	2.7%	1.00 [0.29, 3.49]
Okamoto et al, 2017	81	162	19	38	5.5%	1.00 [0.49, 2.03]
Puzniak et al, 2001	39	602	3	158	3.0%	3.58 [1.09, 11.74]
Reuland et al, 2016	29	202	100	1320	7.6%	2.05 [1.31, 3.18]
Seekatz et al, 2018	13	45	19	86	4.7%	1.43 [0.63, 3.26]
Slaughter et al, 1996	34	101	11	52	5.0%	1.89 [0.86, 4.14]
Søgaard et al, 2017	115	950	224	2779	9.3%	1.57 [1.24, 1.99]
Tan et al, 2018	466	3834	57	1523	9.0%	3.56 [2.68, 4.72]
Wielders et al, 2016	23	288	86	2144	7.3%	2.08 [1.29, 3.35]
**Total (95% CI)**		**9949**		**15701**	**100.0%**	**1.96 [1.54, 2.49]**
Total events	1618		1496			

Heterogeneity: $Tau^2 = 0.15$; $Chi^2 = 65.39$, df = 14 ($P < 0.00001$); $I^2 = 79\%$
Test for overall effect: Z = 5.42 ($P < 0.00001$)

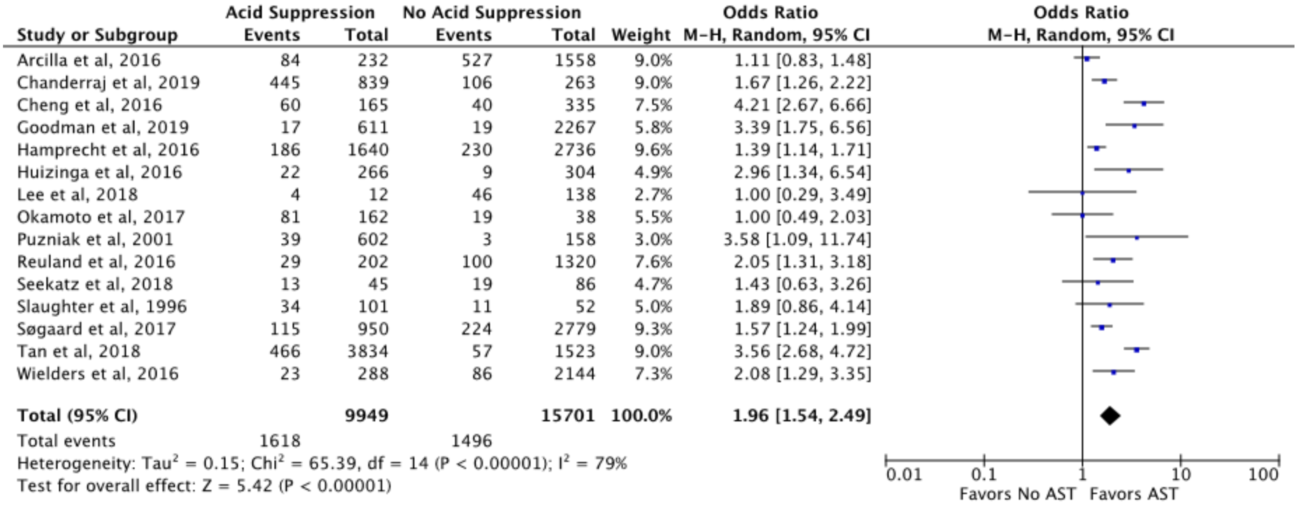

eFigure 7. Funnel Plot

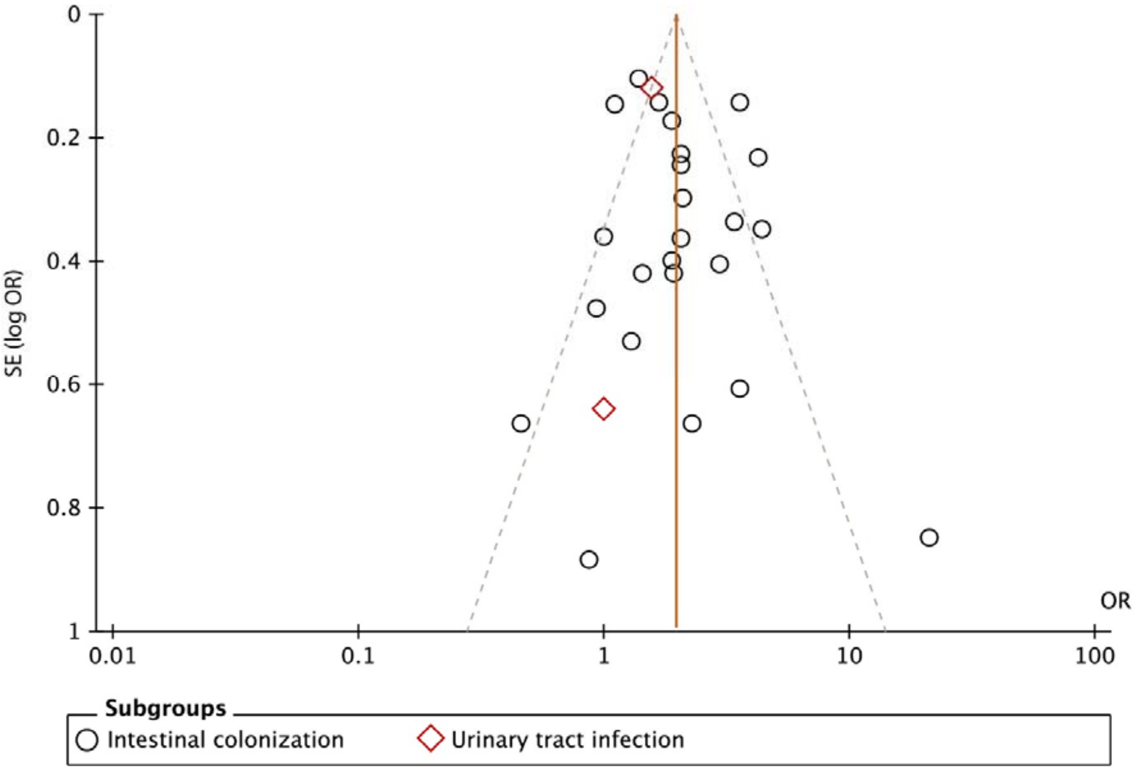

Abbreviations: OR, odds ratio; SE, standard error.
Center orange line represents the summary effect estimate. Outer dotted lines display 95% CIs. Egger's test and Peter's test indicated no publication bias (Egger: *P* = .39; Peter: *P* = .80); publication bias estimators remained unchanged after exclusion of the two studies on the surrogate outcome (Egger: *P* = .40; Peter: *P* = .86). Raw data were used to examine publication bias.

© 2020 Willems RPJ et al. *JAMA Intern Med.*

eReferences

1. Wells GA, Shea B, O'Connell D, et al. The Newcastle-Ottawa Scale (NOS) for assessing the quality of nonrandomized studies in meta-analyses. Ottawa Hospital Research Institute. http://www.ohri.ca/programs/clinical_epidemiology/oxford.asp. Accessed December 12, 2017.

2. World Health Organization. Definition of region groupings. 2013. https://www.who.int/about/who-we-are/regional-offices. Accessed September 5, 2018.

3. Arcilla MS, van Hattem JM, Haverkate MR, et al. Import and spread of extended-spectrum beta-lactamase-producing Enterobacteriaceae by international travellers (COMBAT study): a prospective, multicentre cohort study. *Lancet Infect Dis.* 2017;17(1):78-85.

4. Ben-Ami R, Schwaber MJ, Navon-Venezia S, et al. Influx of extended-spectrum beta-lactamase-producing enterobacteriaceae into the hospital. *Clin Infect Dis.* 2006;42(7):925-934.

5. Chanderraj R, Millar JA, Patel TS, et al. Vancomycin-resistant Enterococcus acquisition in a tertiary care hospital: testing the roles of antibiotic use, proton pump inhibitor use, and colonization pressure. *Open Forum Infect Dis.* 2019;6(4):ofz139.

6. Cheng VC, Chen JH, So SY, et al. A novel risk factor associated with colonization by carbapenemase-producing Enterobacteriaceae: use of proton pump inhibitors in addition to antimicrobial treatment. *Infect Control Hosp Epidemiol.* 2016;37(12):1418-1425.

7. Falk PS, Winnike J, Woodmansee C, Desai M, Mayhall CG. Outbreak of vancomycin-resistant enterococci in a burn unit. *Infect Control Hosp Epidemiol.* 2000;21(9):575-582.

8. Ford CD, Lopansri BK, Haydoura S, et al. Frequency, risk factors, and outcomes of vancomycin-resistant Enterococcus colonization and infection in patients with newly diagnosed acute leukemia: different patterns in patients with acute myelogenous and acute lymphoblastic leukemia. *Infect Control Hosp Epidemiol.* 2015;36(1):47-53.

9. Goodman KE, Simner PJ, Klein EY, et al. Predicting probability of perirectal colonization with carbapenem-resistant Enterobacteriaceae (CRE) and other carbapenem-resistant organisms (CROs) at hospital unit admission. *Infect Control Hosp Epidemiol.* 2019;40(5):541-550.

10. Hagel S, Makarewicz O, Hartung A, et al. ESBL colonization and acquisition in a hospital population: the molecular epidemiology and transmission of resistance genes. *PloS One.* 2019;14(1):e0208505.

11. Hamprecht A, Rohde AM, Behnke M, et al. Colonization with third-generation cephalosporin-resistant Enterobacteriaceae on hospital admission: prevalence and risk factors. *J Antimicrob Chemother.* 2016;71(10):2957-2963.

12. Huizinga P, van den Bergh MK, van Rijen M, Willemsen I, van 't Veer N, Kluytmans J. Proton pump inhibitor use is associated with extended-spectrum beta-lactamase-producing Enterobacteriaceae rectal carriage at hospital admission: a cross-sectional study. *Clin Infect Dis.* 2017;64(3):361-363.

13. Kuenzli E, Jaeger VK, Frei R, et al. High colonization rates of extended-spectrum beta-lactamase (ESBL)-producing Escherichia coli in Swiss travellers to South Asia- a prospective observational multicentre cohort study looking at epidemiology, microbiology and risk factors. *BMC Infect Dis.* 2014;14:528.

14. Latour K, Huang TD, Jans B, et al. Prevalence of multidrug-resistant organisms in nursing homes in Belgium in 2015. *PloS One.* 2019;14(3):e0214327.

15. Lee H, Han SB, Kim JH, Kang S, Durey A. Risk factors of urinary tract infection caused by extended spectrum beta-lactamase-producing Escherichia coli in emergency department. *The Am J Emerg Med.* 2018;36(9):1608-1612.

16. McNeil SA, Malani PN, Chenoweth CE, et al. Vancomycin-resistant enterococcal colonization and infection in liver transplant candidates and recipients: a prospective surveillance study. *Clin Infect Dis.*

© 2020 Willems RPJ et al. *JAMA Intern Med.*

2006;42(2):195-203.

17. Okamoto K, Lin MY, Haverkate M, et al. Modifiable risk factors for the spread of Klebsiella pneumoniae carbapenemase-producing Enterobacteriaceae among long-term acute-care hospital patients. *Infect Control Hosp Epidemiol.* 2017;38(6):670-677.

18. Östholm-Balkhed A, Tarnberg M, Nilsson M, Nilsson LE, Hanberger H, Hallgren A. Travel-associated faecal colonization with ESBL-producing Enterobacteriaceae: incidence and risk factors. *J Antimicrob Chemother.* 2013;68(9):2144-2153.

19. Prasad N, Labaze G, Kopacz J, et al. Asymptomatic rectal colonization with carbapenem-resistant Enterobacteriaceae and Clostridium difficile among residents of a long-term care facility in New York City. *Am J Infect Control.* 2016;44(5):525-532.

20. Puzniak LA, Mayfield J, Leet T, Kollef M, Mundy LM. Acquisition of vancomycin-resistant enterococci during scheduled antimicrobial rotation in an intensive care unit. *Clin Infect Dis.* 2001;33(2):151-157.

21. Reuland EA, Al Naiemi N, Kaiser AM, et al. Prevalence and risk factors for carriage of ESBL-producing Enterobacteriaceae in Amsterdam. *J Antimicrob Chemother*. 2016;71(4):1076-1082.

22. Rodríguez-Baño J, López-Cerero L, Navarro MD, Díaz de Alba P, Pascual A. Faecal carriage of extended-spectrum beta-lactamase-producing Escherichia coli: prevalence, risk factors and molecular epidemiology. *J Antimicrob Chemother*. 2008;62(5):1142-1149.

23. Seekatz AM, Bassis CM, Fogg L, et al. Gut microbiota and clinical features distinguish colonization with Klebsiella pneumoniae carbapenemase-producing Klebsiella pneumoniae at the time of admission to a long-term acute care hospital. *Open Forum Infect Dis.* 2018;5(8):ofy190.

24. Slaughter S, Hayden MK, Nathan C, et al. A comparison of the effect of universal use of gloves and gowns with that of glove use alone on acquisition of vancomycin-resistant enterococci in a medical intensive care unit. *Ann Intern Med.* 1996;125(6):448-456.

25. Søgaard M, Heide-Jorgensen U, Vandenbroucke JP, Schonheyder HC, Vandenbroucke-Grauls C. Risk factors for extended-spectrum beta-lactamase-producing Escherichia coli urinary tract infection in the community in Denmark: a case-control study. *Clin Microbiol Infect*. 2017;23(12):952-960.

26. Tan D, Htun HL, Koh J, et al. Comparative epidemiology of vancomycin-resistant enterococci colonization in an acute-care hospital and its affiliated intermediate- and long-term care facilities in Singapore. *Antimicrob Agents Chemother.* 2018;62(12).

27. Vading M, Kabir MH, Kalin M, et al. Frequent acquisition of low-virulence strains of ESBL-producing Escherichia coli in travellers. *J Antimicrob Chemother.* 2016;71(12):3548-3555.

28. Wielders CCH, van Hoek A, Hengeveld PD, et al. Extended-spectrum beta-lactamase- and pAmpC-producing Enterobacteriaceae among the general population in a livestock-dense area. *Clin Microbiol Infect.* 2017;23(2):120.e121-120.e128.

© 2020 Willems RPJ et al. *JAMA Intern Med.*
